# Supplementary material for: Delivering screening programmes in primary care: protocol for a scoping and systematic mixed studies review
Source: BMJ Open. 2021 Apr 15;11(4):e046331. doi: 10.1136/bmjopen-2020-046331 (PMC8055151; doi:10.1136/bmjopen-2020-046331)
Supplement: Supplementary data [file bmjopen-2020-046331supp001.pdf]

Supplementary file 1: Draft search strategy for primary studies, Ovid MEDLINE

Database: Ovid MEDLINE(R) and Epub Ahead of Print, In-Process & Other Non-Indexed Citations, Daily and Versions(R) <1946 to September 14, 2020>

Search Strategy:

----

1 exp Mass Screening/ or exp Early Diagnosis/ or exp "Early Detection of Cancer"/ or exp undiagnosed diseases/

2 (screening or prevention or (undiagnosed or un-diagnosed)).ti. or (early detection or screening test).mp.

3 (piggyback* or opportunis*).ti,ab.

4 1 or 2 or 3

5 Primary Health Care/ or General Practice/ or Family Practice/ or *General Practitioners/ or *Physicians, Family/ or *Community Health Services/ or *Community Pharmacy Services/ or *Pharmacies/ or *Preventive Health Services/

6 ("primary care" or "primary health*" or "General practi*" or "Family practi*" or "family physician*" or "family doctor*").ti,ab.

7 5 or 6

8 (Implement* or behav* or transl* or embed* or Innovat* or Process* or Run* or Manag* or Plan* or improv* or operat* or organis* or organiz* or integrat* or (identif* adj3 eligi*) or pathway* or central* or deliv* or partner* or evaluat* or diffusion* or effect* or effic* or (quality adj3 control*) or enable* or barrier* or facilitat* or strateg* or interven* or (carry adj out)).ti.

9 *Health Plan Implementation/ or *Implementation Science/ or "Outcome and Process Assessment, Health Care"/ or Program Evaluation/ or *Organizational Innovation/ or *"Delivery of Health Care"/ or *Clinical Audit/ or *Management Audit/ or *Quality Control/

10 8 or 9

11 4 and 7 and 10
