# Supplementary material for: Delivering screening programmes in primary care: protocol for a scoping and systematic mixed studies review
Source: BMJ Open. 2021 Apr 15;11(4):e046331. doi: 10.1136/bmjopen-2020-046331 (PMC8055151; doi:10.1136/bmjopen-2020-046331)
Supplement: Supplementary data [file bmjopen-2020-046331supp003.pdf]

Supplementary file 3: Draft search strategy for best fit framework for research question one, Ovid MEDLINE

Database: Ovid MEDLINE(R) and Epub Ahead of Print, In-Process & Other Non-Indexed Citations, Daily and Versions(R) <1946 to present>

Search Strategy:

----

1 screen*.mp. or exp mass screening/

2 (Pathway* or Process* or Run* or Plan* or Schem* or Blueprint* or Step* or Stage* or Guide*).mp. or exp health plan implementation/ or exp implementation science/ or exp delivery of health care/ [mp=title, abstract, original title, name of substance word, subject heading word, floating sub-heading word, keyword heading word, organism supplementary concept word, protocol supplementary concept word, rare disease supplementary concept word, unique identifier, synonyms]

3 (General practi* or Family practi* or Primary care or Primary health or Family physician* or Family doctor*).mp. or exp General practice/ or exp Primary health care/ or exp Family practice/ or exp General practitioners/ or exp Physicians, family/

4 (pathway* or map or mapping or plan or plans or step or steps or stages or Model* or Theor* or framework* or concept*).mp. or exp Models, theoretical/ or exp Concept formation/

5 1 and 2 and 3 and 4

6 (Regress*model* or integrat* model* or integrat* care model* or economic model* or Markov or Animal or Linear model* or Logistic model* or Likelihood function* or Normogram*).mp. or exp Regression analysis/ or exp Models, statistical/ or exp Markov chains/ or exp Models, chemical/ or exp Models, biological/ or exp Models, molecular/ or exp Fractals/ or exp Metal-organic frameworks/

7 5 not 6
