# Supplementary material for: Delivering screening programmes in primary care: protocol for a scoping and systematic mixed studies review
Source: BMJ Open. 2021 Apr 15;11(4):e046331. doi: 10.1136/bmjopen-2020-046331 (PMC8055151; doi:10.1136/bmjopen-2020-046331)
Supplement: Supplementary data [file bmjopen-2020-046331supp004.pdf]

Supplementary file 4: Draft search strategy for best fit framework for research question two, Ovid MEDLINE

Database: Ovid MEDLINE(R) and Epub Ahead of Print, In-Process & Other Non-Indexed Citations, Daily and Versions(R) <1946 to present>

Search Strategy:

----

1 Screen*.mp. or exp mass screening/

2 (Implement* or deliver* or translat* or integrat* or Innovat* or Manag*).mp. or exp Health plan implementation/ or exp Implementation science/ or exp Organizational innovation/ or exp Diffusion of innovation/ or exp Delivery of health care/

3 (General practi* or Family practi* or Primary care or Primary health or Family physician* or Family doctor*).mp. or exp General practice/ or exp Primary health care/ or exp Family practice/ or exp General practitioners/ or exp Physicians, family/

4 (Model* or Theor* or framework* or concept*).mp. or exp Models, theoretical/ or exp Concept formation/

5 1 and 2 and 3 and 4

6 (Regress*model* or integrat* model* or integrat* care model* or economic model* or Markov or Animal or Linear model* or Logistic model* or Likelihood function* or Normogram*).mp. or exp Regression analysis/ or exp Models, statistical/ or exp Markov chains/ or exp Models, chemical/ or exp Models, biological/ or exp Models, molecular/ or exp Fractals/ or exp Metal-organic frameworks/

7 5 not 6
