# Supplementary material for: Delivering screening programmes in primary care: protocol for a scoping and systematic mixed studies review
Source: BMJ Open. 2021 Apr 15;11(4):e046331. doi: 10.1136/bmjopen-2020-046331 (PMC8055151; doi:10.1136/bmjopen-2020-046331)
Supplement: Author's manuscript [file bmjopen-2020-046331.draft_revisions.pdf]

BMJ Open is committed to open peer review. As part of this commitment we make the peer review history of every article we publish publicly available.

When an article is published we post the peer reviewers' comments and the authors' responses online. We also post the versions of the paper that were used during peer review. These are the versions that the peer review comments apply to.

The versions of the paper that follow are the versions that were submitted during the peer review process. They are not the versions of record or the final published versions. They should not be cited or distributed as the published version of this manuscript.

BMJ Open is an open access journal and the full, final, typeset and author-corrected version of record of the manuscript is available on our site with no access controls, subscription charges or pay-per-view fees (http://bmjopen.bmj.com).

If you have any questions on BMJ Open's open peer review process please email info.bmjopen@bmj.com

BMJ Open

Delivering screening programmes in primary care: protocol for a systematic mixed-studies review

Journal:	*BMJ Open*
Manuscript ID	bmjopen-2020-046331
Article Type:	Protocol
Date Submitted by the Author:	26-Oct-2020

Complete List of Authors:	Modi, Rakesh; University of Cambridge Primary Care Unit,<br>Kelly, Sarah; THIS Institute<br>Hoare, Sarah; THIS Institute<br>Powell, Alison; THIS Institute<br>Kuhn, Isla; University of Cambridge, Medical Library, School of Clinical Medicine<br>Usher-Smith, Juliet; The Primary Care Unit, East Forvie Site<br>Mant, Jonathan; University of Cambridge Primary Care Unit<br>Burt, Jenni; THIS Institute

Keywords:	PRIMARY CARE, PUBLIC HEALTH, PREVENTIVE MEDICINE, STATISTICS & RESEARCH METHODS, Health policy < HEALTH SERVICES ADMINISTRATION & MANAGEMENT

SCHOLARONE™
Manuscripts

I, the Submitting Author has the right to grant and does grant on behalf of all authors of the Work (as defined in the below author licence), an exclusive licence and/or a non-exclusive licence for contributions from authors who are: i) UK Crown employees; ii) where BMJ has agreed a CC-BY licence shall apply, and/or iii) in accordance with the terms applicable for US Federal Government officers or employees acting as part of their official duties; on a worldwide, perpetual, irrevocable, royalty-free basis to BMJ Publishing Group Ltd ("BMJ") its licensees and where the relevant Journal is co-owned by BMJ to the co-owners of the Journal, to publish the Work in this journal and any other BMJ products and to exploit all rights, as set out in our licence.

The Submitting Author accepts and understands that any supply made under these terms is made by BMJ to the Submitting Author unless you are acting as an employee on behalf of your employer or a postgraduate student of an affiliated institution which is paying any applicable article publishing charge ("APC") for Open Access articles. Where the Submitting Author wishes to make the Work available on an Open Access basis (and intends to pay the relevant APC), the terms of reuse of such Open Access shall be governed by a Creative Commons licence – details of these licences and which Creative Commons licence will apply to this Work are set out in our licence referred to above.

Other than as permitted in any relevant BMJ Author's Self Archiving Policies, I confirm this Work has not been accepted for publication elsewhere, is not being considered for publication elsewhere and does not duplicate material already published. I confirm all authors consent to publication of this Work and authorise the granting of this licence.

For peer review only

Delivering screening programmes in primary care: protocol for a systematic mixed-studies review

Authors

1. Dr Rakesh Modi*, rnm30@medschl.cam.ac.uk. Primary Care Unit, University of Cambridge, Strangeways Research Laboratory, Worts Causeway, Cambridge, UK, CB1 8RN
2. Dr Sarah Kelly, sarah.kelly@thisinstitute.cam.ac.uk. The Healthcare Improvement Studies Institute, University of Cambridge, Clifford Allbutt Building, Cambridge Biomedical Campus, Cambridge, UK, CB2 0AH
3. Dr Sarah Hoare, sarah.hoare@thisinstitute.cam.ac.uk. The Healthcare Improvement Studies Institute, University of Cambridge, Clifford Allbutt Building, Cambridge Biomedical Campus, Cambridge, UK, CB2 0AH
4. Dr Alison Powell, alison.powell@thisinstitute.cam.ac.uk. The Healthcare Improvement Studies Institute, University of Cambridge, Clifford Allbutt Building, Cambridge Biomedical Campus, Cambridge, UK, CB2 0AH
5. Ms Isla Kuhn, ilk21@cam.ac.uk. University of Cambridge Medical Library, University of Cambridge School of Clinical Medicine, Box 111, Cambridge Biomedical Campus, Cambridge, UK, CB2 0SP
6. Dr Juliet Usher-Smith, jau20@medschl.cam.ac.uk. Primary Care Unit, University of Cambridge, East Forvie Site, Cambridge Biomedical Campus, Cambridge, UK, CB2 0SR
7. Professor Jonathan Mant, jm677@medschl.cam.ac.uk. Primary Care Unit, University of Cambridge, Strangeways Research Laboratory, Worts Causeway, Cambridge, UK, CB1 8RN
8. Dr Jenni Burt, jenni.burt@thisinstitute.cam.ac.uk. The Healthcare Improvement Studies Institute, University of Cambridge, Clifford Allbutt Building, Cambridge Biomedical Campus, Cambridge, UK, CB2 0AH

* corresponding author

Word count: 4164/4000

ABSTRACT (word count 268/300)

Introduction

Screening programmes represent a considerable amount of health care activity. As complex interventions, they require careful delivery to generate net benefit. Much screening work occurs in primary care. Despite intensive study of intervention delivery in primary care, there is currently no synthesis of the delivery of screening programmes in this setting. The purpose of this review is to describe and critically evaluate the delivery of screening programmes in general practice and community services.

Methods and analysis

We will use scoping review methods to explore which components of screening programmes are delivered in primary care, and systematic review methods to locate and synthesise evidence on how screening programmes can be delivered in primary care, including barriers, facilitators and strategies. We will include empirical studies of any design which consider screening programmes in high-income countries, based in part or whole in primary care. We will search twenty information sources from 1 January 2000, including those relating to health (e.g. MEDLINE, Embase, CINAHL), management (e.g. Rx for change database) and grey literature (e.g. OpenGrey, screening committee websites). Two reviewers will screen citations and full texts of potentially eligible studies, and assess these against inclusion criteria. Qualitative and quantitative data will be extracted in duplicate and synthesised using a Best Fit Framework approach. Within the systematic review,

the Mixed Methods Appraisal Tool will be used to assess risk of bias.

Ethics and dissemination

No ethics approval is required. We will disseminate findings to academics through publication and presentation, to decision-makers through national screening bodies, to practitioners through professional bodies, and to the public through social media.

Registration

PROSPERO registration number: CRD42020215420

KEYWORDS

Screening; delivery; implementation; primary care; systematic review; best fit framework

For peer review only

ARTICLE SUMMARY

Strengths and limitations of this study

- A scoping review followed by a systematic review will together consider a broad spectrum of issues in the delivery of screening programmes in primary care.
- The search of 20 databases and additional key publications will provide a comprehensive basis on which to identify relevant evidence.
- The integration of both quantitative and qualitative data will generate a conceptualisation of screening delivery from multiple paradigms.
- The use of a best fit framework approach will provide accessible answers to the review questions in a diagrammatic format.
- Resource limitations mean that only English language publications have been included in this review.

For peer review only

For peer review only - http://bmjopen.bmj.com/site/about/guidelines.xhtml

INTRODUCTION

Background

Screening aims to identify people with or at risk of a particular condition in an apparently asymptomatic population, with the goal of reducing the risk of ill health related to that condition.[1-3] Official screening programmes account for considerable health spending and activity in high income countries: for example in 2015, the UK National Health Service performed 21 million official screening tests in a population of 65 million.[4-7] These screening programmes typically receive support from the public, professionals and decision-makers, stimulated by the rationale that early detection may prevent ill health.[3 8-10]

Despite positive views, numerous harms of screening are recognised. The identification of participants as having a condition they do not (false positives) and the failed identification of participants who do have the condition (false negatives) is one major source of harm. Harm can also arise through identification of conditions that may never cause illness (overdiagnosis) or treatment of conditions that may never change the outcome (overtreatment).[3 9-12] Screening programmes actively approach people who have not sought medical help, which may have psychosocial consequences.[3 6] The effective and efficient delivery of screening programmes seeks to maximise benefits (including cost-benefits) and minimise harms.[3 7 13]

The delivery of complex healthcare interventions, such as screening, has been the study of multiple disciplines under terms such as ‘implementation science’ and ‘translational research’.[14-16] A number of theories, models and frameworks have been developed to explain and guide the successful delivery of interventions in order to create impact at a reasonable cost.[14 17-19] Initial relatively simple theories of implementation[17 20] have given way to progressively more complex models taking into account multiple layers of agents, networks and dynamics.[14 17 21 22] In parallel, there have been an increasing number of empirical studies of the implementation of health care interventions, and related guidance on how best to design such studies.[14 18 19 21 23-25] To date, however, there has been no synthesis of these studies relevant to the delivery of screening programmes in general.

Primary care, including general practice and community care services, is central to the delivery of many screening programmes,[13 26-31] reflecting its prominent health system role in early detection and disease prevention.[28 32-35] Primary care professionals are familiar with the explanation of results and referrals to specialist care required following screening, and the holistic management of illnesses that might be diagnosed through screening.[29 30 33]

For peer review only - http://bmjopen.bmj.com/site/about/guidelines.xhtml

Health systems that utilise primary care to greater degrees tend to be more cost-effective, and many health systems are further developing the role and nature of primary and community services.[36] As interest in new screening approaches continues to evolve, an expanded role for primary care can be anticipated. However, any new programmes may place additional strain on an already stretched system,[37 38] and evidence on the effective implementation of screening in primary care is required to ensure efficient use of resources.[18]

This review will first scope the literature to consider which aspects of screening programmes have been delivered, or could be delivered, by general practice and community care services. It will then explore how such screening programmes have been delivered in primary care, and the consequences of varying approaches to delivery. Syntheses of evidence will be used to generate recommendations for policy makers, health professionals and researchers of screening programmes. The term 'general practice' is synonymous with family practice and family medicine,[39] and the term 'primary care' refers to general practice and community services.

Objectives

We will review the literature to describe and critically evaluate the delivery of screening programmes in general practice and community services.

We will conduct two linked reviews, answering the following questions:

1. What screening programmes or components of screening programmes have been or could be delivered in general practice and/or community services?
2. How have general practices undertaken the delivery of screening programmes or components of screening programmes, and what has happened as a result of different approaches to delivery?

With question one, our primary objective is to produce a definitive summary of the screening programmes, or components of screening programmes, that general practices and/or community services have delivered, are delivering, or have the potential to deliver. We have included both general practices and community services due to global trends towards integration.[40-43] This will provide important background context for question two, with a primary objective of locating, critically appraising and synthesising the evidence on approaches to the delivery of screening programmes specifically in general practices, including a consideration of the varied systems and strategies used and the barriers, facilitators and outcomes of these. These findings will enable us to generate

For peer review only - http://bmjopen.bmj.com/site/about/guidelines.xhtml

recommendations for the delivery of screening programmes in general practice. Community services were not included in the scope of question two as we felt that the influences on delivery would be too different to those of general practice to provide any meaningful recommendations.

In addition, our focus will be on screening for primary prevention as these mostly involve larger systems, greater coordination and affect more of the population.

METHODS AND ANALYSIS

We will conduct a systematic scoping review to answer question one as the aim is simply to map screening activities that have occurred in primary care.[44] We will conduct a systematic review, including risk of bias assessment, to answer question two.[45] In both reviews, we anticipate locating relevant qualitative and quantitative data: we will use a best-fit framework approach to guide data synthesis (see below).[46-48]

Conduct and reporting

The scoping review for question one will be conducted and reported in accordance with the PRISMA-ScR statement.[44] The systematic review for question two will be conducted and reported in accordance with the PRISMA statement.[49]

This protocol is based on the PRISMA-P guidance[50] and its PROSPERO[51] registration number is CRD42020215420.

Eligibility criteria

Due to the broad scope of these reviews, eligibility criteria have been developed iteratively through the conduct of preliminary searches, examination of records returned, and discussions amongst the authors about areas of contention.

Population

We will include studies that involve primary care institutions, staff, their systems or their actions (e.g. policies, communications etc.). For question one, these will include community services such as pharmacies and outreach programmes. For question two, these will be limited to general practices.

Intervention

For peer review only - http://bmjopen.bmj.com/site/about/guidelines.xhtml

For question one eligible studies will describe the components of screening programmes that have been implemented in primary care. For question two, studies will describe how screening programmes have been delivered in general practice and/or evaluate the different approaches to delivery. For question two, this will include studies exploring barriers, facilitators, systems, strategies or other features that relate to the delivery of screening programmes, or components of screening programmes. External influences such as international policy or patient attitudes may impact on the delivery of screening in general practice: however, studies on such aspects will be included only if they include a specific focus on how these relate to the implementation of screening in general practice. This is to ensure a focus is maintained on components of screening which are under the control of general practice decision-makers.

For both questions, we will focus on screening programmes that are consistent with the general definition that is broadly accepted and stated above: the process of identifying people with or at risk of a particular condition in an apparently asymptomatic population, for the purposes of reducing the risk of ill health related to that condition.[1-3] The key feature is that screening is performed for the purpose of preventing morbidity or mortality. As such, we will only examine those programmes for which there is a clear system for managing the patient following results, from further tests to treatment and follow-up. Polygenic risk scores and new directly marketed screening tests, which provide only an estimate of future disease risk and no clear management plan, will be excluded.[3 52] We will also only include screening programmes that involve systematic invitations to an eligible population and we will exclude programmes that solely rely on opportunistic invitations.[3] We will only study screening programmes for purposes of primary prevention.

Comparator

No comparator group is required.

Phenomena and Outcomes

For question one, included studies will describe the type of screening programmes, or components of these screening programmes, delivered within primary care. Question two is concerned with exploring how screening has been delivered in general practice, and what the results of different approaches to delivery have been. Potential phenomena and outcomes of interest include the systems and strategies used to deliver screening, barriers and facilitators of such approaches, why such approaches were taken, and with what impact. Some studies may provide outcomes such as the level of awareness of screening amongst

For peer review only - http://bmjopen.bmj.com/site/about/guidelines.xhtml

patients, or changes in uptake of screening. If relevant, outcomes may be classified under implementation outcomes such as reach or maintenance.[19 24]

Types of studies

Empirical studies that involve primary data collection or secondary analyses of primary data will be included. We will include all study designs, whether interventional (including randomised controlled trials and quasi-experimental designs), observational (including cohort studies, case-control studies, cross-sectional studies), qualitative, mixed-methods, case reports or case studies. Articles do not need to be peer-reviewed e.g. conference abstracts or grey literature. Studies that do not involve primary empirical research (e.g. reviews, editorials) will be excluded; if found during our search, their reference lists will examined for eligible primary studies. Letters may be included if they contain a primary study.

Setting

Included studies must relate to a primary care setting. In high-income countries, which are of interest to this review, this can be categorised into general practice (health professionals in a local health facility that include family practitioners, who provide some continuous care for all conditions) and community services (other services that provide local health services such as pharmacies and community nursing).

For question one, both general practice and community services are of interest as they are undergoing integration and it will be useful to note their potential in delivering screening programmes. It will also enable us to synthesise a map of screening activities that differentiates their activities. For question two, only general practice will be of interest because issues that affect delivery in general practice were felt likely to be too different to those in community services and therefore a combined synthesis may not provide useful recommendations.

Report characteristics

Studies published from 1 January 2000 onwards will be included to reflect the current role of primary care in the delivery of screening programmes.

Exclusion criteria

- Studies not published in English.
- Secondary research such as reviews and editorials.
- Unpublished material such as personal communications and drafts.

For peer review only - http://bmjopen.bmj.com/site/about/guidelines.xhtml

- Publications that discuss screening delivery but do not clearly base their discussion on primary empirical findings.
- Studies of screening programmes that do not have a clear management plan for results e.g. those that use polygenetic risk scores to provide only an estimate of future disease risk and do not have a clear management plan. Screening for genetic disorders where there is clear management of the patient after results are included e.g. BRCA 1 or 2.
- Studies on screening for secondary or tertiary prevention where a patient is invited because they have a disease that predisposes to the condition that they are being screened for e.g. diabetic retinopathy screening.
- Studies where the participants were not systematically invited for screening, such as opportunistic screening programmes and commercial genetic screening tests.
- Studies on screening delivery in settings other than primary care, such as hospitals or central public health institutions.
- Studies with all data based in low- or middle-income countries, according to the World Bank.[43 53 54]

Information sources

Databases

Table 2 displays the information sources that will be used in their broadest dates of coverage; these were searched in September to November 2020. These sources were chosen after discussions with an information specialist, the review team, and members of the UK National Screening Committee.

For peer review only

For peer review only - http://bmjopen.bmj.com/site/about/guidelines.xhtml

Table 2. Information sources for the review of the delivery of screening programmes in primary care

Databases	Grey literature sources	Other sources
MEDLINE via Ovid	UK NSC website	Author recommended papers
Embase via Ovid	PHE screening blog	UK NSC recommended papers
CINAHL via EbscoHOST	NHS health check website	Reference lists of relevant reviews
Scopus	USPSTF website	
ASSIA via Proquest	OpenGrey	
PsycINFO via EbscoHOST	OpenSIGLE	
	Google Scholar	
	NICE Evidence	
	ICTRP	
	Clinicaltrials.gov	
	Rx for change database	

CINAHL: Cummulative Index of Nursing and Allied Health; ASSIA: Applied Social Sciences Index and Abstracts; ICTRP: WHO International Clinical Trials Registry Platform; UK NSC: UK National Screening Committee; PHE: Public Health England; USPSTF: United States Preventive Services Task Force; NICE: National Institute for Health and Care Excellence

Other sources of publications (see Table 2) will be recommended by the review authors through their experience of research and practice in health screening, and through their initial examination of the results from scoping searches. We have emailed known experts in the UK National Screening Committee, who have published on screening and its implementation.[3] Snowball email contact with further experts suggested by the initial experts, will also be performed as appropriate. We will also employ screening of reference lists of relevant review articles that are retrieved by our search in order to find further eligible primary studies.

Search strategy

A draft search strategy was created by an information specialist (IK), a general practitioner (RM), a health services researcher (JB) and a reviews specialist (SK) with contributions of terms by all authors. Searches were piloted in Ovid MEDLINE, records were examined, key papers were checked for their presence, and through this process the strategy was iteratively refined. The concepts ‘screening’, ‘primary care’ and ‘implementation’ were combined with ‘and’ operators. Each concept consisted of controlled vocabulary terms (e.g. MeSH terms) and free text terms that were combined with ‘or’ operators. The terms will be translated for each database including, where available, the use of controlled vocabulary.

For peer review only - http://bmjopen.bmj.com/site/about/guidelines.xhtml

The same search strategy will be used for both questions one and two. A draft of this in the format for Ovid MEDLINE is shown in Supplementary file 1.

Study records

Data management

We will use Covidence[55] to store and manage records returned from main searches. Other software used will include Endnote,[56] NVivo,[57] Microsoft Word, Microsoft Excel and Microsoft PowerPoint.

Selection process

Records will be downloaded into Covidence for screening. Titles and abstracts of the records returned from the searches, as well as additional publications identified through other sources, will be screened (blinded and in duplicate) by independent reviewers according to the eligibility criteria for both questions one and two. If disagreements between the reviewers cannot be resolved through discussion, a third reviewer will arbitrate the final decision. The full texts of the remaining records will be screened for potential relevance, blinded and in duplicate, according to the same eligibility criteria. Where there is disagreement despite discussion, a third reviewer will arbitrate.

Data and phenomena/outcome items

Although data in this mixed studies review is likely to include unforeseen categories, data extracted for all studies will include:

- Study characteristics: reference, first author, year, funding, country from which data was collected, sample size, intervention/purpose of the study, health condition screened for, primary care staff involved, comparator, data collection method, and study category according to the Mixed Methods Appraisal Tool (MMAT)[58] – 'qualitative,' 'quantitative randomised controlled trials,' 'quantitative non-randomised,' 'quantitative descriptive,' or 'mixed methods.'
- Key demographics, where relevant e.g. age, health status, socioeconomic status.
- Key contextual factors, where relevant e.g. new policies, health emergencies, response to significant events, economic concerns, particular health systems.

Data will be extracted into a Microsoft Excel spreadsheet by two independent reviewers, blinded and in duplicate. Disagreements will be discussed by the two reviewers and arbitrated by a third reviewer if necessary. Data relevant to the review questions will be

For peer review only - http://bmjopen.bmj.com/site/about/guidelines.xhtml

extracted in a similar way and synthesis will then proceed, guided by the best fit framework approach. It is not possible to precisely pre-specify the data available for extraction, and how it will be categorised for synthesis, but we anticipate potential areas may include:

- For question one: setting (general practice, community service), relevant component of a screening programme, related outcome data.
- For question two: phenomena measured (e.g. perceived barriers, effects of audits), types of outcome (qualitative or quantitative), outcome data. Qualitative data extracted will be direct quotes from participants, themes generated by the authors where they are clearly supported by data, and any quantitative data.

Risk of bias in individual studies

For studies included in response to question two, the MMAT tool[58] (see supplementary file 2) will be used to assess the risk of bias at study and outcome level. This will be done by two independent reviewers, blinded and in duplicate. Disagreements will be resolved by discussion, and where agreement cannot be made, a third reviewer will arbitrate.

Data synthesis

The best fit framework approach

Qualitative and quantitative data will undergo a mixed-methods synthesis using a best fit framework approach.[47 48] This approach was developed in response to concerns about the resources required to perform qualitative syntheses using approaches such as framework synthesis, thematic synthesis or meta-ethnography. A framework synthesis may require opinions of the authors that are not always transparent, whilst thematic synthesis or meta-ethnography may require intensive inductive analysis that can be prohibitively time-consuming in large reviews.[48] The best fit framework approach provides a rapid and transparent method for synthesising qualitative data, by using a coding framework built from relevant theories and models identified in the literature. It therefore facilitates an examination of review findings in the light of pre-existing theories (with the potential for further development of these theories) rather than generating theories *de novo*. By combining the deductive methods of framework synthesis (by coding data to *a priori* themes) and inductive methods of meta-ethnography (for data that does not code to a theme in the framework), the best fit framework approach is particularly useful where there are relevant theories in the literature but they have not been refined the particular context of the review question. The method generates a diagrammatic answer to the review question that aims to be easy to understand and clear for policy-makers.

For peer review only - http://bmjopen.bmj.com/site/about/guidelines.xhtml

A best fit framework approach to synthesis thus offers a number of advantages for this review. Notably, theories and models of implementation are available in the literature but have not yet been tested and refined with regards to screening in general. We recognise that best fit framework approaches were originally created for qualitative syntheses, and we are employing a reasonably novel approach of using it for a mixed studies review. However, both qualitative and quantitative data may add depth to and refine a synthesis framework, and combining both is readily straightforward when, as here, quantitative data are likely to be too varied to allow a meaningful meta-analysis.

Searching for models for the best fit frameworks

In this review, a best fit framework will be generated to guide data synthesis for each research question. To produce the initial frameworks, we will systematically search the literature for existing models of relevance to the review questions. These searches will be separate from the main literature search for the review that has been described above. The models may relate to certain core features of the review questions, but not necessarily to the specific context of interest. If multiple models are found, those that are most relevant will be purposively selected and synthesised. For question one, the search will aim to locate publications relevant to creating a pathway of steps in a screening programme. For question two, the search will aim to locate publications relevant to the delivery of screening programmes. We will generate initial search terms using the BeHEMoTh mnemonic (Behaviour of interest, Health context, Exclusions, Models or theories), created to guide systematic searches for theories.[48 59] We will translate initial terms into suitable search strings for two main databases: EBSCO CINAHL and Ovid MEDLINE (see supplementary file 3 for Ovid MEDLINE search terms for question one, and supplementary file 4 for question two).

Relevant located models will be chosen using an adaptation of the approach outlined in the original description of the best fit framework method[48] and the BeHEMoTH search strategy.[59] As with the original best fit framework approach, we will conduct systematic searches to locate relevant models. However, we will expand the search with controlled vocabulary terms, add key papers suggested by the research team, and we will purposively select final included models to increase variation in concepts and to represent the different levels of delivery in screening programmes (e.g. public health management and general practice staff). We will also draw on relevant models known from the grey literature.[48]

Synthesis using the best fit frameworks

For peer review only - http://bmjopen.bmj.com/site/about/guidelines.xhtml

In this way, best fit frameworks will be produced separately for question one and question two. The final frameworks (i.e. the outcomes of the searches for models), whether representing a single pre-existing model, or a synthesis of more than one pre-existing model, will subsequently be used as a framework for coding data extracted from included studies.[47] Extracted quantitative and qualitative data will be coded against the best fit framework concepts. Relationships between concepts that emerge from the data will also be noted and will help understand how concepts relate to each other. Where there is not an appropriate concept, a new code will be created. This code will either be an original code or an existing code from another model encountered during the search for the best fit framework. These new codes might undergo concurrent thematic analysis, leading to refinement of existing concepts in the best fit framework, the creation of new concepts, or the alteration of relationships between them, as appropriate. The concepts of the framework that have become redundant after this process may be removed.[48] For each review question, this will therefore produce a framework that has been refined by qualitative data and described by quantitative data, such as ranges for effect sizes of the concepts. By using the relationships between concepts uncovered in this process, a new model that displays these relationships will be produced to answer each review question. Data, codes, themes and models will be discussed in regular team meetings, which will actively encourage analyses through different perspectives and assumptions, contributing to the robustness of the findings. An overview of the incorporation of the best fit framework approach in this review is shown in figure 1.

The authors of the original best fit framework method recommended conducting a sensitivity analysis by removing low quality studies.[48] However, this is not appropriate for this review: assessment of the quality of qualitative studies is often only based on the quality of reporting, and it is widely recognised that for complex interventions[60] even 'low quality' studies may provide useful indications of relevant concepts that need further exploration.

Meta-biases and confidence in cumulative evidence

Publication bias will not be assessed due to the variety of evidence types in this review and its lesser usefulness in a complex exploratory review such as this. However, by including grey literature and databases that include conference abstracts, we hope to limit this.[61] We will reflect and comment on individual study risk of biases, recurrent types of bias and potential effects of these on the overall conclusions. The strength of recommendation may be described with a GRADE score informed by the MMAT scores of individual studies that address the same recommendation. However, due to the variety of studies that may help answer the review question, such a score may not prove useful; in this case, this process will not be undertaken.

For peer review only - http://bmjopen.bmj.com/site/about/guidelines.xhtml

Patient and public involvement

Patients and public were not involved in the design or writing of this publication as this review will mainly generate lessons for health care providers and decision makers. We have, therefore, included authors in these roles. We do, however, intend to disseminate the results of the review via social media and directly to relevant contacts in patient organisations and primary care bodies.

ETHICS AND DISSEMINATION

All data is available in the public domain so ethical approval is not required. If major amendments are required, the nature, rationale and date will be documented in PROSPERO. Outputs will be published in peer reviewed journals and presented at international conference. Publications will be shared through reviewers' and institutions' social media, networks, and websites, thus reaching academics, policy makers and the public. They will also be sent to members of various national screening committees and primary care bodies through established links.

ACKNOWLEDGEMENTS

We are grateful to Professor Anne Mackie and Ms Katharine Thompson of Public Health England, and Mr Matthew Fagg of the NHS Diabetes Programme for advising on key grey literature related to screening delivery in primary care.

COMPETING INTERESTS

RM, SH, AP, JM and JB are undertaking a trial of Atrial Fibrillation Screening (SAFER trial) funded by the National Institute for Health Research.

FUNDING

RM's work for this review is supported by the Wellcome Trust as part of the Wellcome Trust PhD Programme for Primary Care Clinicians [grant number RHZB/195]. SK, IK and JB are supported by the Health Foundation's grant to the University of Cambridge for The Healthcare Improvement Studies (THIS) Institute. SH and AP contributed to this publications through independent research funded by the National Institute for Health Research (NIHR) under its Programme Grants for Applied Research Programme (Reference Number RP-PG-0217-20007). JUS and JM are funded by the University of Cambridge. JM is an NIHR Senior Investigator. All the funders had no involvement in the development of this protocol and will have no involvement in any aspect of the review itself. The views expressed are

For peer review only - http://bmjopen.bmj.com/site/about/guidelines.xhtml

those of the author(s) and not necessarily those of the NHS, the Wellcome Trust, the NIHR or the Department of Health.

CONTRIBUTIONS

JB is the guarantor. RM drafted the manuscript. All authors contributed to eligibility criteria, and review methods. RM, JB, IK and SK created the search strategy. JUS, SH, AP and JB provided expertise on screening. All authors edited and approved the final manuscript.

REFERENCES

1. World Health Organization. Screening: World Health Organization; 2019 [Available from: https://www.who.int/cancer/prevention/diagnosis-screening/screening/en/ accessed 16/12/2019.
2. Public Health England. NHS population screening explained: Public Health England; 2013 [updated 17/07/2019. Available from: https://www.gov.uk/guidance/nhs-population-screening-explained accessed 16/12/2019.
3. Raffle AE, Mackie A, Gray JAM. Screening: Evidence and Practice. second ed. Oxford, United Kingdom: Oxford University Press 2019:14-15.
4. Public Health England. NHS Screening Programmes in England: Public Health England, 2016.
5. Office for National Statistics. Population estimates for the UK, England and Wales, Scotland and Northern Ireland: mid-2015: Office for National Statistics; 2016 [Available from: https://www.ons.gov.uk/peoplepopulationandcommunity/populationandmigration/populationestimates/bulletins/annualmidyearpopulationestimates/mid2015 accessed 16/12/2019.
6. Armstrong N, Eborall H. The sociology of medical screening: past, present and future. *Sociology of Health & Illness* 2012;34(2):161-76. doi: 10.1111/j.1467-9566.2011.01441.x
7. Richards M. Report of the independent review of adult screening programmes in England: NHS England, 2019.
8. Schwartz LM, Woloshin S, Fowler J, Floyd J., et al. Enthusiasm for Cancer Screening in the United States. *Jama* 2004;291(1):71-78. doi: 10.1001/jama.291.1.71
9. Woolf SH, Harris R. The Harms of Screening: New Attention to an Old Concern. *Jama* 2012;307(6):565-66. doi: 10.1001/jama.2012.100
10. Hoffmann TC, Del Mar C. Patients' Expectations of the Benefits and Harms of Treatments, Screening, and Tests: A Systematic Review. *JAMA Internal Medicine* 2015;175(2):274-86. doi: 10.1001/jamainternmed.2014.6016
11. Hoffmann TC, Del Mar C. Clinicians' Expectations of the Benefits and Harms of Treatments, Screening, and Tests: A Systematic Review. *JAMA Internal Medicine* 2017;177(3):407-19. doi: 10.1001/jamainternmed.2016.8254
12. Wegwarth O, Gigerenzer G. Overdiagnosis and Overtreatment: Evaluation of What Physicians Tell Their Patients About Screening Harms. *JAMA Internal Medicine* 2013;173(22):2086-88. doi: 10.1001/jamainternmed.2013.10363
13. Seedat F, Cooper J, Cameron L, et al. International comparisons of screening policy-making: A systematic review 2014.
14. Braithwaite J, Churruca K, Long JC, et al. When complexity science meets implementation science: a theoretical and empirical analysis of systems change. *BMC Medicine* 2018;16(1):63. doi: 10.1186/s12916-018-1057-z
15. Elmore RF. Backward Mapping: Implementation Research and Policy Decisions. *Political Science Quarterly* 1979;94(4):601-16. doi: 10.2307/2149628

For peer review only - http://bmjopen.bmj.com/site/about/guidelines.xhtml

16. Noble CH. The Eclectic Roots of Strategy Implementation Research. *Journal of Business Research* 1999;45(2):119-34. doi: https://doi.org/10.1016/S0148-2963(97)00231-2
17. Lanham HJ, Leykum LK, Taylor BS, et al. How complexity science can inform scale-up and spread in health care: understanding the role of self-organization in variation across local contexts. *Social science & medicine (1982)* 2013;93:194-202. doi: 10.1016/j.socscimed.2012.05.040 [published Online First: 2012/07/24]
18. Bauer MS, Damschroder L, Hagedorn H, et al. An introduction to implementation science for the non-specialist. *BMC psychology* 2015;3:32. doi: 10.1186/s40359-015-0089-9 [published Online First: 2015/09/18]
19. Peters DH, Adam T, Alonge O, et al. Implementation research: what it is and how to do it. *BMJ : British Medical Journal* 2013;347:f6753. doi: 10.1136/bmj.f6753
20. Greenhalgh T, Papoutsi C. Studying complexity in health services research: desperately seeking an overdue paradigm shift. *BMC Medicine* 2018;16(1):95. doi: 10.1186/s12916-018-1089-4
21. Long KM, McDermott F, Meadows GN. Being pragmatic about healthcare complexity: our experiences applying complexity theory and pragmatism to health services research. *BMC Medicine* 2018;16(1):94. doi: 10.1186/s12916-018-1087-6
22. Plsek PE, Wilson T. Complexity, leadership, and management in healthcare organisations. *BMJ (Clinical research ed)* 2001;323(7315):746-49. doi: 10.1136/bmj.323.7315.746
23. Cohn S, Clinch M, Bunn C, et al. Entangled complexity: why complex interventions are just not complicated enough. *Journal of health services research & policy* 2013;18(1):40-3. doi: 10.1258/jhsrp.2012.012036 [published Online First: 2013/02/09]
24. Proctor E, Silmere H, Raghavan R, et al. Outcomes for implementation research: conceptual distinctions, measurement challenges, and research agenda. *Administration and policy in mental health* 2011;38(2):65-76. doi: 10.1007/s10488-010-0319-7 [published Online First: 2010/10/20]
25. Davies P, Walker AE, Grimshaw JM. A systematic review of the use of theory in the design of guideline dissemination and implementation strategies and interpretation of the results of rigorous evaluations. *Implementation Science* 2010;5(1):14. doi: 10.1186/1748-5908-5-14
26. Robson J. Screening in general practice and primary care. *British Medical Bulletin* 1998;54(4):961-82. doi: 10.1093/oxfordjournals.bmb.a011741
27. Lai CK, Igarashi A, Lau NM, et al. Cancer screening for older people: to screen or not to screen. *Hong Kong medical journal = Xianggang yi xue za zhi* 2017;23(5):503-16. doi: 10.12809/hkmj166154 [published Online First: 2017/09/02]
28. Shimizu T, Bouchard M, Mavriplis C. Update on age-appropriate preventive measures and screening for Canadian primary care providers. *Can Fam Physician* 2016;62(2):131-38.
29. Emery JD, Shaw K, Williams B, et al. The role of primary care in early detection and follow-up of cancer. *Nature Reviews Clinical Oncology* 2014;11(1):38-48. doi: 10.1038/nrclinonc.2013.212
30. Miser WF. Cancer screening in the primary care setting: the role of the primary care physician in screening for breast, cervical, colorectal, lung, ovarian, and prostate cancers. *Primary care* 2007;34(1):137-67. doi: 10.1016/j.pop.2007.02.002 [published Online First: 2007/05/08]
31. Frame PS, Werth PL. How primary health care providers can integrate cancer prevention into practice. *Cancer* 1993;72(3 Suppl):1132-7. doi: 10.1002/1097-0142(19930801)72:3+<1132::aid-cncr2820721331>3.0.co;2-v [published Online First: 1993/08/01]
32. Kale MS, Korenstein D. Overdiagnosis in primary care: framing the problem and finding solutions. *BMJ (Clinical research ed)* 2018;362:k2820-k20. doi: 10.1136/bmj.k2820
33. Simonian SJ. Screening and Identification in Pediatric Primary Care. *Behavior Modification* 2006;30(1):114-31. doi: 10.1177/0145445505283311
34. Hamilton W, Walter FM, Rubin G, et al. Improving early diagnosis of symptomatic cancer. *Nature reviews Clinical oncology* 2016;13(12):740-49. doi: 10.1038/nrclinonc.2016.109 [published Online First: 2016/11/03]

For peer review only - http://bmjopen.bmj.com/site/about/guidelines.xhtml

35. World Health Organization. Primary Health Care: World Health Organization; 2019 [updated 27/02/2019. Available from: https://www.who.int/news-room/fact-sheets/detail/primary-health-care accessed 27/11/2019.
36. Macinko J, Starfield B, Shi L. The Contribution of Primary Care Systems to Health Outcomes within Organization for Economic Cooperation and Development (OECD) Countries, 1970–1998. *Health Services Research* 2003;38(3):831-65. doi: 10.1111/1475-6773.00149
37. World Health Organization. The World Health Report 2008 - primary Health Care (Now More Than Ever). Geneva, Switzerland: World Health Organization, 2008.
38. Rao M, Pilot E. The missing link--the role of primary care in global health. *Glob Health Action* 2014;7:23693. doi: 10.3402/gha.v7.23693 [published Online First: 2014/02/25]
39. Allen J, Gay B, Crebolder H, et al. The European definition of general practice / family medicine: WONCA Europe, 2005.
40. WHO Regional Office for Europe. Integrated care models: an overview: working document. Copenhagen, Denmark: World Health Organization, 2016.
41. O'Gurek DT, Czapp P, Lampton L, et al. Integration of Primary Care and Public Health (Position Paper): American Academy of Family Physicians, 2015.
42. Baird B. Primary care networks explained: The King's Fund; 2019 [updated 01/07/2019. Available from: https://www.kingsfund.org.uk/publications/primary-care-networks-explained accessed 11/03/2020.
43. Mounier-Jack S, Mayhew SH, Mays N. Integrated care: learning between high-income, and low-and middle-income country health systems. *Health Policy and Planning* 2017;32(suppl_4):iv6-iv12. doi: 10.1093/heapol/czx039
44. Tricco AC, Lillie E, Zarin W, et al. PRISMA Extension for Scoping Reviews (PRISMA-ScR): Checklist and Explanation. *Annals of Internal Medicine* 2018;169(7):467-73. doi: 10.7326/m18-0850
45. Centre for Reviews and Disseminaton. Systematic Reviews: CRD's guidance for undertaking reviews in health care. York, United Kingdom: Centre for Reviews and Dissemination, University of York, 2009.
46. Noyes J, Booth A, Cargo M, et al. Chapter 21: Qualitative evidence. In: Cochrane Handbook for Systematic Reviews of Interventions version 6.0. In: Higgins J, Thomas J, Chandler J, et al., eds.: Cochrane, 2019.
47. Carroll C, Booth A, Cooper K. A worked example of "best fit" framework synthesis: A systematic review of views concerning the taking of some potential chemopreventive agents. *BMC Medical Research Methodology* 2011;11(1):29. doi: 10.1186/1471-2288-11-29
48. Carroll C, Booth A, Leaviss J, et al. "Best fit" framework synthesis: refining the method. *BMC Medical Research Methodology* 2013;13(1):37. doi: 10.1186/1471-2288-13-37
49. Moher D, Liberati A, Tetzlaff J, et al. Preferred reporting items for systematic reviews and meta-analyses: the PRISMA statement. *BMJ* 2009;339:b2535. doi: 10.1136/bmj.b2535
50. Moher D, Shamseer L, Clarke M, et al. Preferred reporting items for systematic review and meta-analysis protocols (PRISMA-P) 2015 statement. *Systematic reviews* 2015;4(1):1.
51. PROSPERO. PROSPERO: International prospective register of systematic reviews 2020 [Available from: https://www.crd.york.ac.uk/prospero/ accessed 16/03/2020.
52. Scott IA, Attia J, Moynihan R. Promises and perils of using genetic tests to predict risk of disease. *BMJ* 2020;368:m14. doi: 10.1136/bmj.m14
53. WHO Evaluation Office. Review of 40 years of primary health care implementation at country level: World Health Organization, 2019.
54. The World Bank. World Bank Country and Lending Groups: The World Bank; 2019 [Available from: https://datahelpdesk.worldbank.org/knowledgebase/articles/906519-world-bank-country-and-lending-groups accessed 27/11/2019.
55. Covidence. Covidence: Better systematic review management 2020 [Available from: https://www.covidence.org/home accessed 19/06/2020.
56. Clarivate. Endnote 2020 [Available from: https://endnote.com/14/08/2020.

For peer review only - http://bmjopen.bmj.com/site/about/guidelines.xhtml

57. QSR International. NVivo 2020 [Available from: https://www.qsrinternational.com/nvivo-qualitative-data-analysis-software/home accessed 19/06/2020.
58. Hong Q, Pluye P, Fàbregues S, et al. Mixed Methods Appraisal Tool (MMAT), version 2018. Registration of Copyright (#1148552), Canadian Intellectual Property Office, Industry Canada., 2018.
59. Booth A, Carroll C. Systematic searching for theory to inform systematic reviews: is it feasible? Is it desirable? *Health Information & Libraries Journal* 2015;32(3):220-35.
60. Dixon-Woods M, Shaw Rl Fau - Agarwal S, Agarwal S Fau - Smith JA, et al. The problem of appraising qualitative research. (1475-3898 (Print))
61. Petticrew M, Egan M, Thomson H, et al. Publication bias in qualitative research: what becomes of qualitative research presented at conferences? *Journal of Epidemiology and Community Health* 2008;62(6):552-54. doi: 10.1136/jech.2006.059394

FIGURES

Figure 1. Explanation of the role of best fit frameworks in the syntheses for the scoping and systematic reviews on the delivery of screening programmes in primary care.

For peer review only

For peer review only - http://bmjopen.bmj.com/site/about/guidelines.xhtml

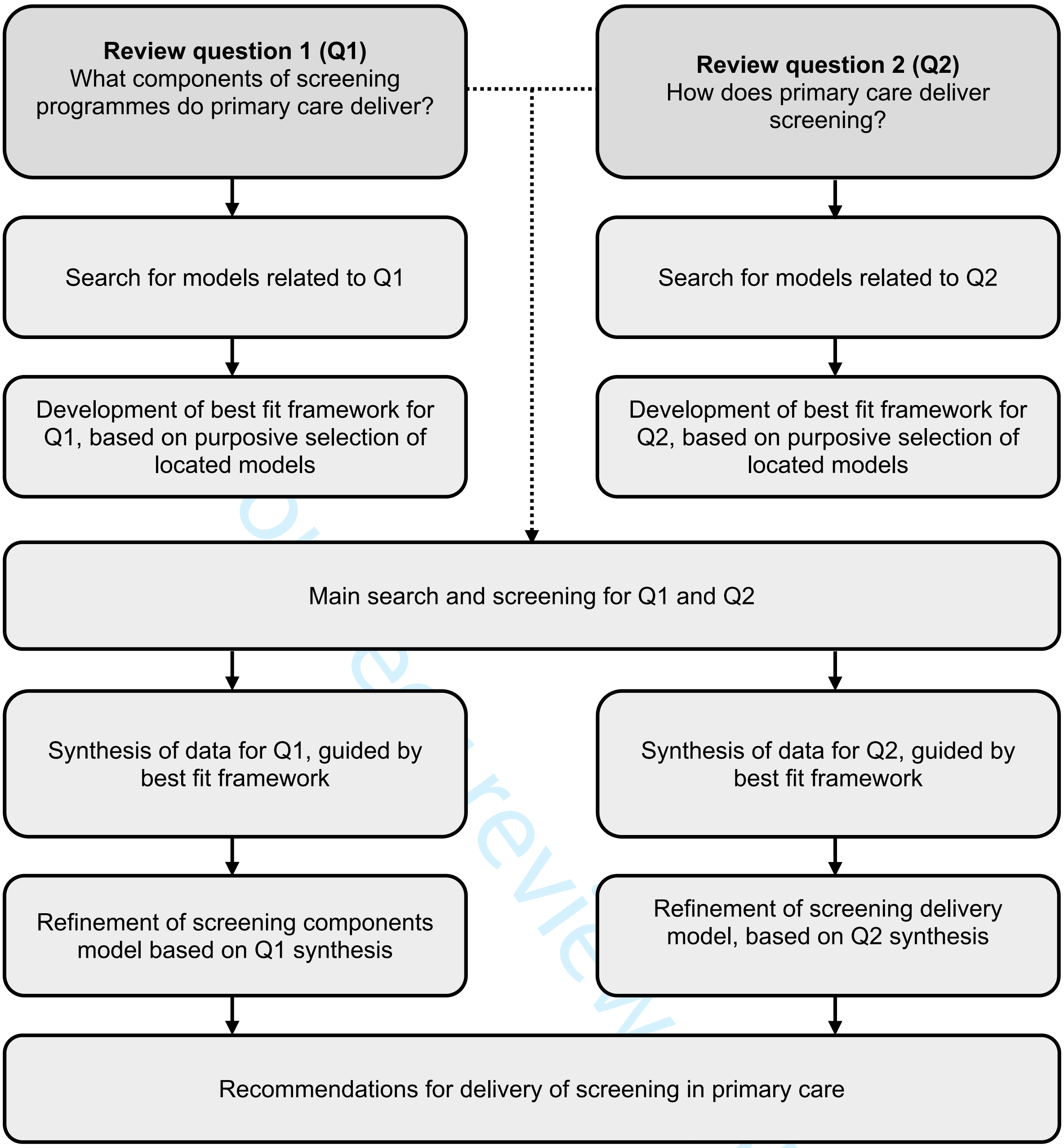
Review question 1 (Q1)
What components of screening programmes do primary care deliver?
Review question 2 (Q2)
How does primary care deliver screening?
Search for models related to Q1
Search for models related to Q2
Development of best fit framework for Q1, based on purposive selection of located models
Development of best fit framework for Q2, based on purposive selection of located models
Main search and screening for Q1 and Q2
Synthesis of data for Q1, guided by best fit framework
Synthesis of data for Q2, guided by best fit framework
Refinement of screening components model based on Q1 synthesis
Refinement of screening delivery model, based on Q2 synthesis
Recommendations for delivery of screening in primary care

Supplementary file 1: Draft search strategy for primary studies, Ovid MEDLINE

Database: Ovid MEDLINE(R) and Epub Ahead of Print, In-Process & Other Non-Indexed Citations, Daily and Versions(R) <1946 to September 14, 2020>

Search Strategy:

----

1 exp Mass Screening/ or exp Early Diagnosis/ or exp "Early Detection of Cancer"/ or exp undiagnosed diseases/

2 (screening or prevention or (undiagnosed or un-diagnosed)).ti. or (early detection or screening test).mp.

3 (piggyback* or opportunis*).ti,ab.

4 1 or 2 or 3

5 Primary Health Care/ or General Practice/ or Family Practice/ or *General Practitioners/ or *Physicians, Family/ or *Community Health Services/ or *Community Pharmacy Services/ or *Pharmacies/ or *Preventive Health Services/

6 ("primary care" or "primary health*" or "General practi*" or "Family practi*" or "family physician*" or "family doctor*").ti,ab.

7 5 or 6

8 (Implement* or behav* or transl* or embed* or Innovat* or Process* or Run* or Manag* or Plan* or improv* or operat* or organis* or organiz* or integrat* or (identif* adj3 eligi*) or pathway* or central* or deliv* or partner* or evaluat* or diffusion* or effect* or effic* or (quality adj3 control*) or enable* or barrier* or facilitat* or strateg* or interven* or (carry adj out)).ti.

9 *Health Plan Implementation/ or *Implementation Science/ or "Outcome and Process Assessment, Health Care"/ or Program Evaluation/ or *Organizational Innovation/ or *"Delivery of Health Care"/ or *Clinical Audit/ or *Management Audit/ or *Quality Control/

10 8 or 9

11 4 and 7 and 10

For peer review only

Supplementary file 2: MMAT checklist from Hong et al. 2018[1]

Part I: Mixed Methods Appraisal Tool (MMAT), version 2018

Category of study designs	Methodological quality criteria	Responses			
		Yes	No	Can't tell	Comments
Screening questions (for all types)	S1. Are there clear research questions?				
	S2. Do the collected data allow to address the research questions?				
	Further appraisal may not be feasible or appropriate when the answer is 'No' or 'Can't tell' to one or both screening questions.				
1. Qualitative	1.1. Is the qualitative approach appropriate to answer the research question?				
	1.2. Are the qualitative data collection methods adequate to address the research question?				
	1.3. Are the findings adequately derived from the data?				
	1.4. Is the interpretation of results sufficiently substantiated by data?				
	1.5. Is there coherence between qualitative data sources, collection, analysis and interpretation?				
2. Quantitative randomized controlled trials	2.1. Is randomization appropriately performed?				
	2.2. Are the groups comparable at baseline?				
	2.3. Are there complete outcome data?				
	2.4. Are outcome assessors blinded to the intervention provided?				
	2.5 Did the participants adhere to the assigned intervention?				
3. Quantitative non-randomized	3.1. Are the participants representative of the target population?				
	3.2. Are measurements appropriate regarding both the outcome and intervention (or exposure)?				
	3.3. Are there complete outcome data?				
	3.4. Are the confounders accounted for in the design and analysis?				
	3.5. During the study period, is the intervention administered (or exposure occurred) as intended?				
4. Quantitative descriptive	4.1. Is the sampling strategy relevant to address the research question?				
	4.2. Is the sample representative of the target population?				
	4.3. Are the measurements appropriate?				
	4.4. Is the risk of nonresponse bias low?				
	4.5. Is the statistical analysis appropriate to answer the research question?				
5. Mixed methods	5.1. Is there an adequate rationale for using a mixed methods design to address the research question?				
	5.2. Are the different components of the study effectively integrated to answer the research question?				
	5.3. Are the outputs of the integration of qualitative and quantitative components adequately interpreted?				
	5.4. Are divergences and inconsistencies between quantitative and qualitative results adequately addressed?				
	5.5. Do the different components of the study adhere to the quality criteria of each tradition of the methods involved?				

1. Hong Q, Pluye P, Fàbregues S, et al. Mixed Methods Appraisal Tool (MMAT), version 2018. Registration of Copyright (#1148552), Canadian Intellectual Property Office, Industry Canada., 2018.

For peer review only

Supplementary file 3: Draft search strategy for best fit framework for research question one, Ovid MEDLINE

Database: Ovid MEDLINE(R) and Epub Ahead of Print, In-Process & Other Non-Indexed Citations, Daily and Versions(R) <1946 to present>

Search Strategy:

----

1 screen*.mp. or exp mass screening/

2 (Pathway* or Process* or Run* or Plan* or Schem* or Blueprint* or Step* or Stage* or Guide*).mp. or exp health plan implementation/ or exp implementation science/ or exp delivery of health care/ [mp=title, abstract, original title, name of substance word, subject heading word, floating sub-heading word, keyword heading word, organism supplementary concept word, protocol supplementary concept word, rare disease supplementary concept word, unique identifier, synonyms]

3 (General practi* or Family practi* or Primary care or Primary health or Family physician* or Family doctor*).mp. or exp General practice/ or exp Primary health care/ or exp Family practice/ or exp General practitioners/ or exp Physicians, family/

4 (pathway* or map or mapping or plan or plans or step or steps or stages or Model* or Theor* or framework* or concept*).mp. or exp Models, theoretical/ or exp Concept formation/

5 1 and 2 and 3 and 4

6 (Regress*model* or integrat* model* or integrat* care model* or economic model* or Markov or Animal or Linear model* or Logistic model* or Likelihood function* or Normogram*).mp. or exp Regression analysis/ or exp Models, statistical/ or exp Markov chains/ or exp Models, chemical/ or exp Models, biological/ or exp Models, molecular/ or exp Fractals/ or exp Metal-organic frameworks/

7 5 not 6

For peer review only

Supplementary file 4: Draft search strategy for best fit framework for research question two, Ovid MEDLINE

Database: Ovid MEDLINE(R) and Epub Ahead of Print, In-Process & Other Non-Indexed Citations, Daily and Versions(R) <1946 to present>

Search Strategy:

----

1 Screen*.mp. or exp mass screening/

2 (Implement* or deliver* or translat* or integrat* or Innovat* or Manag*).mp. or exp Health plan implementation/ or exp Implementation science/ or exp Organizational innovation/ or exp Diffusion of innovation/ or exp Delivery of health care/

3 (General practi* or Family practi* or Primary care or Primary health or Family physician* or Family doctor*).mp. or exp General practice/ or exp Primary health care/ or exp Family practice/ or exp General practitioners/ or exp Physicians, family/

4 (Model* or Theor* or framework* or concept*).mp. or exp Models, theoretical/ or exp Concept formation/

5 1 and 2 and 3 and 4

6 (Regress*model* or integrat* model* or integrat* care model* or economic model* or Markov or Animal or Linear model* or Logistic model* or Likelihood function* or Normogram*).mp. or exp Regression analysis/ or exp Models, statistical/ or exp Markov chains/ or exp Models, chemical/ or exp Models, biological/ or exp Models, molecular/ or exp Fractals/ or exp Metal-organic frameworks/

7 5 not 6

For peer review only

PRISMA-P (Preferred Reporting Items for Systematic review and Meta-Analysis Protocols) 2015 checklist: recommended items to address in a systematic review protocol*

Section and topic	Item No	Checklist item	
**ADMINISTRATIVE INFORMATION**			
Title:			
Identification	1a	Identify the report as a protocol of a systematic review	title page
Update	1b	If the protocol is for an update of a previous systematic review, identify as such	N/A
Registration	2	If registered, provide the name of the registry (such as PROSPERO) and registration number	Abstract
Authors:			
Contact	3a	Provide name, institutional affiliation, e-mail address of all protocol authors; provide physical mailing address of corresponding author	Title page
Contributions	3b	Describe contributions of protocol authors and identify the guarantor of the review	p12
Amendments	4	If the protocol represents an amendment of a previously completed or published protocol, identify as such and list changes; otherwise, state plan for documenting important protocol amendments	p11
Support:			
Sources	5a	Indicate sources of financial or other support for the review	p12
Sponsor	5b	Provide name for the review funder and/or sponsor	p12
Role of sponsor or funder	5c	Describe roles of funder(s), sponsor(s), and/or institution(s), if any, in developing the protocol	p12
**INTRODUCTION**			
Rationale	6	Describe the rationale for the review in the context of what is already known	p1-2
Objectives	7	Provide an explicit statement of the question(s) the review will address with reference to participants, interventions, comparators, and outcomes (PICO)	p2 (PICO format not appropriate)
**METHODS**			
Eligibility criteria	8	Specify the study characteristics (such as PICO, study design, setting, time frame) and report characteristics (such as years considered, language, publication status) to be used as criteria for eligibility for the review	p3-6
Information sources	9	Describe all intended information sources (such as electronic databases, contact with study authors, trial registers or other grey literature sources) with planned dates of coverage	

For peer review only - http://bmjopen.bmj.com/site/about/guidelines.xhtml

			p6-7
Search strategy	10	Present draft of search strategy to be used for at least one electronic database, including planned limits, such that it could be repeated	p7-8 and supplementary file 1
Study records:			
Data management	11a	Describe the mechanism(s) that will be used to manage records and data throughout the review	p8
Selection process	11b	State the process that will be used for selecting studies (such as two independent reviewers) through each phase of the review (that is, screening, eligibility and inclusion in meta-analysis)	p8
Data collection process	11c	Describe planned method of extracting data from reports (such as piloting forms, done independently, in duplicate), any processes for obtaining and confirming data from investigators	p8-9
Data items	12	List and define all variables for which data will be sought (such as PICO items, funding sources), any pre-planned data assumptions and simplifications	p8-9
Outcomes and prioritization	13	List and define all outcomes for which data will be sought, including prioritization of main and additional outcomes, with rationale	p9
Risk of bias in individual studies	14	Describe anticipated methods for assessing risk of bias of individual studies, including whether this will be done at the outcome or study level, or both; state how this information will be used in data synthesis	p9 and supplementary file 2
Data synthesis	15a	Describe criteria under which study data will be quantitatively synthesised	p9-11
	15b	If data are appropriate for quantitative synthesis, describe planned summary measures, methods of handling data and methods of combining data from studies, including any planned exploration of consistency (such as I^2, Kendall's τ)	N/A
	15c	Describe any proposed additional analyses (such as sensitivity or subgroup analyses, meta-regression)	p11
	15d	If quantitative synthesis is not appropriate, describe the type of summary planned	p9-11
Meta-bias(es)	16	Specify any planned assessment of meta-bias(es) (such as publication bias across studies, selective reporting within studies)	p11
Confidence in cumulative evidence	17	Describe how the strength of the body of evidence will be assessed (such as GRADE)	p11

*** It is strongly recommended that this checklist be read in conjunction with the PRISMA-P Explanation and Elaboration (cite when available) for important clarification on the items. Amendments to a review protocol should be tracked and dated. The copyright for PRISMA-P (including checklist) is held by the PRISMA-P Group and is distributed under a Creative Commons Attribution Licence 4.0.**

For peer review only

From: Shamseer L, Moher D, Clarke M, Ghersi D, Liberati A, Petticrew M, Shekelle P, Stewart L, PRISMA-P Group. Preferred reporting items for systematic review and meta-analysis protocols (PRISMA-P) 2015: elaboration and explanation. BMJ. 2015 Jan 2;349(jan02 1):g7647.

For peer review only

BMJ Open

Delivering screening programmes in primary care: protocol for a scoping and systematic mixed-studies review

Journal:	*BMJ Open*
Manuscript ID	bmjopen-2020-046331.R1
Article Type:	Protocol
Date Submitted by the Author:	24-Mar-2021

Complete List of Authors:	Modi, Rakesh; University of Cambridge Primary Care Unit,<br>Kelly, Sarah; THIS Institute<br>Hoare, Sarah; THIS Institute<br>Powell, Alison; THIS Institute<br>Kuhn, Isla; University of Cambridge, Medical Library, School of Clinical Medicine<br>Usher-Smith, Juliet; The Primary Care Unit, East Forvie Site<br>Mant, Jonathan; University of Cambridge Primary Care Unit<br>Burt, Jenni; THIS Institute

**Primary Subject Heading**:	General practice / Family practice
Secondary Subject Heading:	Public health, Health services research, Health policy
Keywords:	PRIMARY CARE, PUBLIC HEALTH, PREVENTIVE MEDICINE, STATISTICS & RESEARCH METHODS, Health policy < HEALTH SERVICES ADMINISTRATION & MANAGEMENT

SCHOLARONE™ Manuscripts

I, the Submitting Author has the right to grant and does grant on behalf of all authors of the Work (as defined in the below author licence), an exclusive licence and/or a non-exclusive licence for contributions from authors who are: i) UK Crown employees; ii) where BMJ has agreed a CC-BY licence shall apply, and/or iii) in accordance with the terms applicable for US Federal Government officers or employees acting as part of their official duties; on a worldwide, perpetual, irrevocable, royalty-free basis to BMJ Publishing Group Ltd ("BMJ") its licensees and where the relevant Journal is co-owned by BMJ to the co-owners of the Journal, to publish the Work in this journal and any other BMJ products and to exploit all rights, as set out in our licence.

The Submitting Author accepts and understands that any supply made under these terms is made by BMJ to the Submitting Author unless you are acting as an employee on behalf of your employer or a postgraduate student of an affiliated institution which is paying any applicable article publishing charge ("APC") for Open Access articles. Where the Submitting Author wishes to make the Work available on an Open Access basis (and intends to pay the relevant APC), the terms of reuse of such Open Access shall be governed by a Creative Commons licence – details of these licences and which Creative Commons licence will apply to this Work are set out in our licence referred to above.

Other than as permitted in any relevant BMJ Author's Self Archiving Policies, I confirm this Work has not been accepted for publication elsewhere, is not being considered for publication elsewhere and does not duplicate material already published. I confirm all authors consent to publication of this Work and authorise the granting of this licence.

For peer review only

Delivering screening programmes in primary care: protocol for a scoping and systematic mixed-studies review

Authors

1. Dr Rakesh Modi*, rnm30@medschl.cam.ac.uk. Primary Care Unit, University of Cambridge, Strangeways Research Laboratory, Worts Causeway, Cambridge, UK, CB1 8RN
2. Dr Sarah Kelly, sarah.kelly@thisinstitute.cam.ac.uk. The Healthcare Improvement Studies Institute, University of Cambridge, Clifford Allbutt Building, Cambridge Biomedical Campus, Cambridge, UK, CB2 0AH
3. Dr Sarah Hoare, sarah.hoare@thisinstitute.cam.ac.uk. The Healthcare Improvement Studies Institute, University of Cambridge, Clifford Allbutt Building, Cambridge Biomedical Campus, Cambridge, UK, CB2 0AH
4. Dr Alison Powell, alison.powell@thisinstitute.cam.ac.uk. The Healthcare Improvement Studies Institute, University of Cambridge, Clifford Allbutt Building, Cambridge Biomedical Campus, Cambridge, UK, CB2 0AH
5. Ms Isla Kuhn, ilk21@cam.ac.uk. University of Cambridge Medical Library, University of Cambridge School of Clinical Medicine, Box 111, Cambridge Biomedical Campus, Cambridge, UK, CB2 0SP
6. Dr Juliet Usher-Smith, jau20@medschl.cam.ac.uk. Primary Care Unit, University of Cambridge, East Forvie Site, Cambridge Biomedical Campus, Cambridge, UK, CB2 0SR
7. Professor Jonathan Mant, jm677@medschl.cam.ac.uk. Primary Care Unit, University of Cambridge, Strangeways Research Laboratory, Worts Causeway, Cambridge, UK, CB1 8RN
8. Dr Jenni Burt, jenni.burt@thisinstitute.cam.ac.uk. The Healthcare Improvement Studies Institute, University of Cambridge, Clifford Allbutt Building, Cambridge Biomedical Campus, Cambridge, UK, CB2 0AH

* corresponding author

Word count: 4214/4000

For peer review only - http://bmjopen.bmj.com/site/about/guidelines.xhtml

ABSTRACT (word count 268/300)

Introduction

Screening programmes represent a considerable amount of health care activity. As complex interventions, they require careful delivery to generate net benefit. Much screening work occurs in primary care. Despite intensive study of intervention delivery in primary care, there is currently no synthesis of the delivery of screening programmes in this setting. The purpose of this review is to describe and critically evaluate the delivery of screening programmes in general practice and community services.

Methods and analysis

We will use scoping review methods to explore which components of screening programmes are delivered in primary care, and systematic review methods to locate and synthesise evidence on how screening programmes can be delivered in primary care, including barriers, facilitators and strategies. We will include empirical studies of any design which consider screening programmes in high-income countries, based in part or whole in primary care. We will search twenty information sources from 1 January 2000, including those relating to health (e.g. MEDLINE, Embase, CINAHL), management (e.g. Rx for change database) and grey literature (e.g. OpenGrey, screening committee websites). Two reviewers will screen citations and full texts of potentially eligible studies, and assess these against inclusion criteria. Qualitative and quantitative data will be extracted in duplicate and synthesised using a Best Fit Framework approach. Within the systematic review,
the Mixed Methods Appraisal Tool will be used to assess risk of bias.

Ethics and dissemination

No ethics approval is required. We will disseminate findings to academics through publication and presentation, to decision-makers through national screening bodies, to practitioners through professional bodies, and to the public through social media.

Registration

PROSPERO registration number: CRD42020215420

KEYWORDS

Screening; delivery; implementation; primary care; systematic review; best fit framework

For peer review only

ARTICLE SUMMARY

Strengths and limitations of this study

- A scoping review followed by a systematic review will together consider a broad spectrum of issues in the delivery of screening programmes in primary care.
- The search of 20 databases and additional key publications will provide a comprehensive basis on which to identify relevant evidence.
- The integration of both quantitative and qualitative data will generate a conceptualisation of screening delivery from multiple paradigms.
- The use of a best fit framework approach will provide accessible answers to the review questions in a diagrammatic format.
- Resource limitations mean that only English language publications have been included in this review.

For peer review only

For peer review only - http://bmjopen.bmj.com/site/about/guidelines.xhtml

INTRODUCTION

Background

Screening aims to identify people with or at risk of a particular condition in an apparently asymptomatic population, with the goal of reducing the risk of ill health related to that condition.[1-3] Official screening programmes, such as breast cancer screening or newborn bloodspot testing, account for considerable health spending and activity in high income countries: for example in 2015, the UK National Health Service performed 21 million official screening tests in a population of 65 million.[4-7] These screening programmes typically receive support from the public, professionals and decision-makers, stimulated by the rationale that early detection may prevent ill health.[3 8-10]

Despite positive views, numerous harms of screening are recognised. The identification of participants as having a condition they do not (false positives) and the failed identification of participants who do have the condition (false negatives) is one major source of harm. Harm can also arise through identification of conditions that may never cause illness (overdiagnosis) or treatment of conditions that may never change the outcome (overtreatment).[3 9-12] Screening programmes actively approach people who have not sought medical help, which may have psychosocial consequences.[3 6] The effective and efficient delivery of screening programmes seeks to maximise benefits (including cost-benefits) and minimise harms.[3 7 13]

The delivery of complex healthcare interventions, such as screening, has been the study of multiple disciplines under terms such as 'implementation science' and 'translational research'.[14-16] A number of theories, models and frameworks have been developed to explain and guide the successful delivery of interventions in order to create impact at a reasonable cost.[14 17-19] Initial relatively simple theories of implementation[17 20] have given way to progressively more complex models taking into account multiple layers of agents, networks and dynamics.[14 17 21 22] In parallel, there have been an increasing number of empirical studies of the implementation of health care interventions, and related guidance on how best to design such studies.[14 18 19 21 23-25] To date, however, there has been no synthesis of these studies relevant to the delivery of screening programmes in general.

Primary care, including general practice and community care services, is central to the delivery of many screening programmes (e.g. cervical screening, general health checks),[13 26-31] reflecting its prominent health system role in early detection and disease prevention.[28 32-35] Primary care professionals are familiar with the explanation of results and referrals to

For peer review only - http://bmjopen.bmj.com/site/about/guidelines.xhtml

specialist care required following screening, and the holistic management of illnesses that might be diagnosed through screening.[29 30 33] Health systems that utilise primary care to greater degrees tend to be more cost-effective, and many health systems are further developing the role and nature of primary and community services.[36] As interest in new screening approaches continues to evolve, an expanded role for primary care can be anticipated. However, any new programmes may place additional strain on an already stretched system,[37 38] and evidence on the effective implementation of screening in primary care is required to ensure efficient use of resources.[18]

This review will first scope the literature to consider which aspects of screening programmes have been delivered, or could be delivered, by general practice and community care services. It will then explore how such screening programmes have been delivered in primary care, and the consequences of varying approaches to delivery. Syntheses of evidence will be used to generate recommendations for policy makers, health professionals and researchers of screening programmes. The term 'general practice' is synonymous with family practice and family medicine,[39] and the term 'primary care' refers to general practice and community services.

Objectives

We will review the literature to describe and critically evaluate the delivery of screening programmes in general practice and community services.

We will conduct two linked reviews, answering the following questions:

1. What screening programmes or components of screening programmes have been or could be delivered in general practice and/or community services?
2. How have general practices undertaken the delivery of screening programmes or components of screening programmes, and what has happened as a result of different approaches to delivery?

With question one, our primary objective is to produce a definitive summary of the screening programmes, or components of screening programmes, that general practices and/or community services have delivered, are delivering, or have the potential to deliver. We have included both general practices and community services due to global trends towards integration.[40-43] This will provide important background context for question two, with a primary objective of locating, critically appraising and synthesising the evidence on approaches to the delivery of screening programmes specifically in general practices, including a consideration of the varied systems and strategies used and the barriers,

For peer review only - http://bmjopen.bmj.com/site/about/guidelines.xhtml

facilitators and outcomes of these. These findings will enable us to generate recommendations for the delivery of screening programmes in general practice. Community services were not included in the scope of question two as we felt that the influences on delivery would be too different to those of general practice to provide any meaningful recommendations.

In addition, our focus will be on screening for primary prevention as these mostly involve larger systems, greater coordination and affect more of the population.

METHODS AND ANALYSIS

We will conduct a systematic scoping review to answer question one as the aim is simply to map screening activities that have occurred in primary care.[44] We will conduct a systematic review, including risk of bias assessment, to answer question two.[45] In both reviews, we anticipate locating relevant qualitative and quantitative data: we will use a best-fit framework approach to guide data synthesis (see below).[46-48]

Conduct and reporting

The scoping review for question one will be conducted and reported in accordance with the PRISMA-ScR statement.[44] The systematic review for question two will be conducted and reported in accordance with the PRISMA statement.[49]

This protocol is based on the PRISMA-P guidance[50] and its PROSPERO[51] registration number is CRD42020215420.

Eligibility criteria

Due to the broad scope of these reviews, eligibility criteria have been developed iteratively through the conduct of preliminary searches, examination of records returned, and discussions amongst the authors about areas of contention.

Population

We will include studies that involve primary care institutions, staff, their systems or their actions (e.g. policies, communications etc.). For question one, these will include community services such as pharmacies and outreach programmes. For question two, these will be limited to general practices.

Intervention

For peer review only - http://bmjopen.bmj.com/site/about/guidelines.xhtml

For peer review only

For question one eligible studies will describe the components of screening programmes that have been implemented in primary care. For question two, studies will describe how screening programmes have been delivered in general practice and/or evaluate the different approaches to delivery. For question two, this will include studies exploring barriers, facilitators, systems, strategies or other features that relate to the delivery of screening programmes, or components of screening programmes. External influences such as international policy or patient attitudes may impact on the delivery of screening in general practice: however, studies on such aspects will be included only if they include a specific focus on how these relate to the implementation of screening in general practice. This is to ensure a focus is maintained on components of screening which are under the control of general practice decision-makers.

For both questions, we will focus on screening programmes that are consistent with the general definition that is broadly accepted and stated above: the process of identifying people with or at risk of a particular condition in an apparently asymptomatic population, for the purposes of reducing the risk of ill health related to that condition.[1-3] The key feature is that screening is performed for the purpose of preventing morbidity or mortality. As such, we will only examine those programmes for which there is a clear system for managing the patient following results, from further tests to treatment and follow-up. Polygenic risk scores and new directly marketed screening tests, which provide only an estimate of future disease risk and no clear management plan, will be excluded.[3 52] We will also only include screening programmes that involve systematic invitations to an eligible population and we will exclude programmes that solely rely on opportunistic invitations.[3] We will only study screening programmes for purposes of primary prevention.

Comparator

No comparator group is required.

Phenomena and Outcomes

For question one, included studies will describe the type of screening programmes, or components of these screening programmes, delivered within primary care. Question two is concerned with exploring how screening has been delivered in general practice, and what the results of different approaches to delivery have been. Potential phenomena and outcomes of interest include the systems and strategies used to deliver screening, barriers and facilitators of such approaches, and why such approaches were taken. Some studies will also report impacts of these phenomena and outcomes. These are not essential to be

For peer review only - http://bmjopen.bmj.com/site/about/guidelines.xhtml

For peer review only

included and could be clinical (e.g. changes in mortality) or implementation impacts (e.g. changes in attitudes, reach or maintenance).[19 24]

Types of studies

Empirical studies that involve primary data collection or secondary analyses of primary data will be included. We will include all study designs, whether interventional (including randomised controlled trials and quasi-experimental designs), observational (including cohort studies, case-control studies, cross-sectional studies), qualitative, mixed-methods, case reports or case studies. Articles do not need to be peer-reviewed e.g. conference abstracts or grey literature. Studies that do not involve primary empirical research (e.g. reviews, editorials) will be excluded; if found during our search, their reference lists will examined for eligible primary studies. Letters may be included if they contain a primary study.

Setting

Included studies must relate to a primary care setting. In high-income countries, which are of interest to this review, this can be categorised into general practice (health professionals in a local health facility that include family practitioners, who provide some continuous care for all conditions) and community services (other services that provide local health services such as pharmacies and community nursing).

For question one, both general practice and community services are of interest as they are undergoing integration and it will be useful to note their potential in delivering screening programmes. It will also enable us to synthesise a map of screening activities that differentiates their activities. For question two, only general practice will be of interest because issues that affect delivery in general practice were felt likely to be too different to those in community services and therefore a combined synthesis may not provide useful recommendations.

Report characteristics

Studies published from 1 January 2000 onwards will be included to reflect the current role of primary care in the delivery of screening programmes.

Exclusion criteria

- Studies not published in English.
- Secondary research such as reviews and editorials.
- Unpublished material such as personal communications and drafts.

For peer review only - http://bmjopen.bmj.com/site/about/guidelines.xhtml

- Publications that discuss screening delivery but do not clearly base their discussion on primary empirical findings.
- Studies of screening programmes that do not have a clear management plan for results e.g. those that use polygenetic risk scores to provide only an estimate of future disease risk and do not have a clear management plan. Screening for genetic disorders where there is clear management of the patient after results are included e.g. BRCA 1 or 2.
- Studies on screening for secondary or tertiary prevention where a patient is invited because they have a disease that predisposes to the condition that they are being screened for e.g. diabetic retinopathy screening.
- Studies where the participants were not systematically invited for screening, such as opportunistic screening programmes and commercial genetic screening tests.
- Studies on screening delivery in settings other than primary care, such as hospitals or central public health institutions.
- Studies with all data based in low- or middle-income countries, according to the World Bank.[43 53 54]

Information sources

Databases

Table 1 displays the information sources that will be used in their broadest dates of coverage; these were searched in September to November 2020. These sources were chosen after discussions with an information specialist, the review team, and members of the UK National Screening Committee.

For peer review only - http://bmjopen.bmj.com/site/about/guidelines.xhtml

Table 1. Information sources for the review of the delivery of screening programmes in primary care

Databases	Grey literature sources	Other sources
MEDLINE via Ovid	UK NSC website	Author recommended papers
Embase via Ovid	PHE screening blog	UK NSC recommended papers
CINAHL via EbscoHOST	NHS health check website	Reference lists of relevant reviews
Scopus	USPSTF website	
ASSIA via Proquest	OpenGrey	
PsycINFO via EbscoHOST	OpenSIGLE	
	Google Scholar	
	NICE Evidence	
	ICTRP	
	Clinicaltrials.gov	
	Rx for change database	

CINAHL: Cummulative Index of Nursing and Allied Health; ASSIA: Applied Social Sciences Index and Abstracts; ICTRP: WHO International Clinical Trials Registry Platform; UK NSC: UK National Screening Committee; PHE: Public Health England; USPSTF: United States Preventive Services Task Force; NICE: National Institute for Health and Care Excellence

Other sources of publications (see Table 1) will be recommended by the review authors through their experience of research and practice in health screening, and through their initial examination of the results from scoping searches. We have emailed known experts in the UK National Screening Committee, who have published on screening and its implementation.[3] Snowball email contact with further experts suggested by the initial experts, will also be performed as appropriate. We will also employ screening of reference lists of relevant review articles that are retrieved by our search in order to find further eligible primary studies.

Search strategy

A draft search strategy was created by an information specialist (IK), a general practitioner (RM), a health services researcher (JB) and a reviews specialist (SK) with contributions of terms by all authors. Searches were piloted in all research databases employed in this review, records were examined, key papers were checked for their presence, and through this process the strategy was iteratively refined. The concepts ‘screening’, ‘primary care’ and ‘implementation’ were combined with ‘and’ operators. Each concept consisted of controlled vocabulary terms (e.g. MeSH terms) and free text terms that were combined with ‘or’ operators. The terms will be translated for each database including, where available, the use

For peer review only - http://bmjopen.bmj.com/site/about/guidelines.xhtml

of controlled vocabulary. The same search strategy will be used for both questions one and two. A draft of this in the format for Ovid MEDLINE is shown in Supplementary file 1.

Study records

Data management

We will use Covidence[55] to store and manage records returned from main searches. Other software used will include Endnote,[56] NVivo,[57] Microsoft Word, Microsoft Excel and Microsoft PowerPoint.

Selection process

Records will be downloaded into Covidence for screening. Titles and abstracts of the records returned from the searches, as well as additional publications identified through other sources, will be screened (blinded and in duplicate) by independent reviewers according to the eligibility criteria for both questions one and two. If disagreements between the reviewers cannot be resolved through discussion, a third reviewer will arbitrate the final decision. The full texts of the remaining records will be screened for potential relevance, blinded and in duplicate, according to the same eligibility criteria. Where there is disagreement despite discussion, a third reviewer will arbitrate.

Data and phenomena/outcome items

Although data in this mixed studies review is likely to include unforeseen categories, data extracted for all studies will include:

- Study characteristics: reference, first author, year, funding, country from which data was collected, sample size, intervention/purpose of the study, health condition screened for, primary care staff involved, comparator, data collection method, and study category according to the Mixed Methods Appraisal Tool (MMAT)[58] – 'qualitative,' 'quantitative randomised controlled trials,' 'quantitative non-randomised,' 'quantitative descriptive,' or 'mixed methods.'
- Key demographics, where relevant e.g. age, health status, socioeconomic status.
- Key contextual factors, where relevant e.g. new policies, health emergencies, response to significant events, economic concerns, particular health systems.

Data will be extracted into a Microsoft Excel spreadsheet by two independent reviewers, blinded and in duplicate. Disagreements will be discussed by the two reviewers and arbitrated by a third reviewer if necessary. Data relevant to the review questions will be

For peer review only - http://bmjopen.bmj.com/site/about/guidelines.xhtml

extracted in a similar way and synthesis will then proceed, guided by the best fit framework approach. It is not possible to precisely pre-specify the data available for extraction, and how it will be categorised for synthesis, but we anticipate potential areas may include:

- For question one: setting (general practice, community service), relevant component of a screening programme, related outcome data.
- For question two: phenomena measured (e.g. perceived barriers, effects of audits), types of outcome (qualitative or quantitative), outcome data. Qualitative data extracted will be direct quotes from participants, themes generated by the authors where they are clearly supported by data, and any quantitative data.

Risk of bias in individual studies

For studies included in response to question two, the MMAT tool[58] (see supplementary file 2) will be used to assess the risk of bias at study and outcome level. This will be done by two independent reviewers, blinded and in duplicate. Disagreements will be resolved by discussion, and where agreement cannot be made, a third reviewer will arbitrate.

Data synthesis

The best fit framework approach

Qualitative and quantitative data will undergo a mixed-methods synthesis using a best fit framework approach.[47 48] This approach was developed in response to concerns about the resources required to perform qualitative syntheses using approaches such as framework synthesis, thematic synthesis or meta-ethnography. A framework synthesis may require opinions of the authors that are not always transparent, whilst thematic synthesis or meta-ethnography may require intensive inductive analysis that can be prohibitively time-consuming in large reviews.[48] The best fit framework approach provides a rapid and transparent method for synthesising qualitative data, by using a coding framework built from relevant theories and models identified in the literature. It therefore facilitates an examination of review findings in the light of pre-existing theories (with the potential for further development of these theories) rather than generating theories *de novo*. By combining the deductive methods of framework synthesis (by coding data to *a priori* themes) and inductive methods of meta-ethnography (for data that does not code to a theme in the framework), the best fit framework approach is particularly useful where there are relevant theories in the literature but they have not been refined the particular context of the review question. The method generates a diagrammatic answer to the review question that aims to be easy to understand and clear for policy-makers.

For peer review only - http://bmjopen.bmj.com/site/about/guidelines.xhtml

A best fit framework approach to synthesis thus offers a number of advantages for this review. Notably, theories and models of implementation are available in the literature but have not yet been tested and refined with regards to screening in general. We recognise that best fit framework approaches were originally created for qualitative syntheses, and we are employing a reasonably novel approach of using it for a mixed studies review. However, both qualitative and quantitative data may add depth to and refine a synthesis framework, and combining both is readily straightforward when, as here, quantitative data are likely to be too varied to allow a meaningful meta-analysis.

Searching for models for the best fit frameworks

In this review, a best fit framework will be generated to guide data synthesis for each research question. To produce the initial frameworks, we will systematically search the literature for existing models of relevance to the review questions. These searches will be separate from the main literature search for the review that has been described above. The models may relate to certain core features of the review questions, but not necessarily to the specific context of interest. If multiple models are found, those that are most relevant will be purposively selected and synthesised. For question one, the search will aim to locate publications relevant to creating a pathway of steps in a screening programme. For question two, the search will aim to locate publications relevant to the delivery of screening programmes. We will generate initial search terms using the BeHEMoTh mnemonic (Behaviour of interest, Health context, Exclusions, Models or theories), created to guide systematic searches for theories.[48 59] We will translate initial terms into suitable search strings for two main databases: EBSCO CINAHL and Ovid MEDLINE (see supplementary file 3 for Ovid MEDLINE search terms for question one, and supplementary file 4 for question two).

Relevant located models will be chosen using an adaptation of the approach outlined in the original description of the best fit framework method[48] and the BeHEMoTH search strategy.[59] As with the original best fit framework approach, we will conduct systematic searches to locate relevant models. However, we will expand the search with controlled vocabulary terms, add key papers suggested by the research team, and we will purposively select final included models to increase variation in concepts and to represent the different levels of delivery in screening programmes (e.g. public health management and general practice staff). We will also draw on relevant models known from the grey literature.[48]

Synthesis using the best fit frameworks

For peer review only - http://bmjopen.bmj.com/site/about/guidelines.xhtml

In this way, best fit frameworks will be produced separately for question one and question two. The final frameworks (i.e. the outcomes of the searches for models), whether representing a single pre-existing model, or a synthesis of more than one pre-existing model, will subsequently be used as a framework for coding data extracted from included studies.[47] Extracted quantitative and qualitative data will be coded against the best fit framework concepts. Relationships between concepts that emerge from the data will also be noted and will help understand how concepts relate to each other. Where there is not an appropriate concept, a new code will be created. This code will either be an original code or an existing code from another model encountered during the search for the best fit framework. These new codes might undergo concurrent thematic analysis, leading to refinement of existing concepts in the best fit framework, the creation of new concepts, or the alteration of relationships between them, as appropriate. The concepts of the framework that have become redundant after this process may be removed.[48] For each review question, this will therefore produce a framework that has been refined by qualitative data and described by quantitative data, which will be narratively synthesised where there is sufficient information. By using the relationships between concepts uncovered in this process, a new model that displays these relationships will be produced to answer each review question. Data, codes, themes and models will be discussed in regular team meetings, which will actively encourage analyses through different perspectives and assumptions, contributing to the robustness of the findings. An overview of the incorporation of the best fit framework approach in this review is shown in figure 1.

The authors of the original best fit framework method recommended conducting a sensitivity analysis by removing low quality studies.[48] However, this is not appropriate for this review: assessment of the quality of qualitative studies is often only based on the quality of reporting, and it is widely recognised that for complex interventions[60] even ‘low quality’ studies may provide useful indications of relevant concepts that need further exploration. We will, however, use the risk of bias assessment to inform our interpretation of findings for review question two.

Meta-biases and confidence in cumulative evidence

Publication bias will not be assessed due to the variety of evidence types in this review and its lesser usefulness in a complex exploratory review such as this. However, by including grey literature and databases that include conference abstracts, we hope to limit this.[61] We will reflect and comment on individual study risk of biases, recurrent types of bias and potential effects of these on the overall conclusions for review question two, including how they might create differences in study findings. The strength of recommendation may be

For peer review only - http://bmjopen.bmj.com/site/about/guidelines.xhtml

described with a GRADE score informed by the MMAT scores of individual studies that address the same recommendation. However, due to the variety of studies that may help answer the review question, such a score may not prove useful; in this case, this process will not be undertaken.

Patient and public involvement

Patients and public were not involved in the design or writing of this publication as this review will mainly generate lessons for health care providers and decision makers. We have, therefore, included authors in these roles. We do, however, intend to disseminate the results of the review via social media and directly to relevant contacts in patient organisations and primary care bodies.

ETHICS AND DISSEMINATION

All data is available in the public domain so ethical approval is not required. If major amendments are required, the nature, rationale and date will be documented in PROSPERO. Outputs will be published in peer reviewed journals and presented at international conference. Publications will be shared through reviewers' and institutions' social media, networks, and websites, thus reaching academics, policy makers and the public. They will also be sent to members of various national screening committees and primary care bodies through established links.

ACKNOWLEDGEMENTS

We are grateful to Professor Anne Mackie and Ms Katharine Thompson of Public Health England, and Mr Matthew Fagg of the NHS Diabetes Programme for advising on key grey literature related to screening delivery in primary care.

COMPETING INTERESTS

RM, SH, AP, JM and JB are undertaking a trial of Atrial Fibrillation Screening (SAFER trial) funded by the National Institute for Health Research.

FUNDING

RM's work for this review is supported by the Wellcome Trust as part of the Wellcome Trust PhD Programme for Primary Care Clinicians [grant number 203921/Z/16/Z]. SK, IK and JB are supported by the Health Foundation's grant to the University of Cambridge for The Healthcare Improvement Studies (THIS) Institute. SH and AP contributed to this publications through independent research funded by the National Institute for Health Research (NIHR) under its Programme Grants for Applied Research Programme (Reference Number RP-PG-

For peer review only - http://bmjopen.bmj.com/site/about/guidelines.xhtml

0217-20007). JUS and JM are funded by the University of Cambridge. JM is an NIHR Senior Investigator. All the funders had no involvement in the development of this protocol and will have no involvement in any aspect of the review itself. The views expressed are those of the author(s) and not necessarily those of the NHS, the Wellcome Trust, the NIHR or the Department of Health.

CONTRIBUTIONS

JB is the guarantor. RM drafted the manuscript. All authors contributed to eligibility criteria, and review methods. RM, JB, IK and SK created the search strategy. JUS, SH, AP, JM and JB provided expertise on screening. All authors edited and approved the final manuscript.

REFERENCES

1. World Health Organization. Screening: World Health Organization; 2019 [Available from: https://www.who.int/cancer/prevention/diagnosis-screening/screening/en/ accessed 16/12/2019.
2. Public Health England. NHS population screening explained: Public Health England; 2013 [updated 17/07/2019. Available from: https://www.gov.uk/guidance/nhs-population-screening-explained accessed 16/12/2019.
3. Raffle AE, Mackie A, Gray JAM. Screening: Evidence and Practice. second ed. Oxford, United Kingdom: Oxford University Press 2019:14-15.
4. Public Health England. NHS Screening Programmes in England: Public Health England, 2016.
5. Office for National Statistics. Population estimates for the UK, England and Wales, Scotland and Northern Ireland: mid-2015: Office for National Statistics; 2016 [Available from: https://www.ons.gov.uk/peoplepopulationandcommunity/populationandmigration/populationestimates/bulletins/annualmidyearpopulationestimates/mid2015 accessed 16/12/2019.
6. Armstrong N, Eborall H. The sociology of medical screening: past, present and future. *Sociology of Health & Illness* 2012;34(2):161-76. doi: 10.1111/j.1467-9566.2011.01441.x
7. Richards M. Report of the independent review of adult screening programmes in England: NHS England, 2019.
8. Schwartz LM, Woloshin S, Fowler J, Floyd J., et al. Enthusiasm for Cancer Screening in the United States. *Jama* 2004;291(1):71-78. doi: 10.1001/jama.291.1.71
9. Woolf SH, Harris R. The Harms of Screening: New Attention to an Old Concern. *Jama* 2012;307(6):565-66. doi: 10.1001/jama.2012.100
10. Hoffmann TC, Del Mar C. Patients' Expectations of the Benefits and Harms of Treatments, Screening, and Tests: A Systematic Review. *JAMA Internal Medicine* 2015;175(2):274-86. doi: 10.1001/jamainternmed.2014.6016
11. Hoffmann TC, Del Mar C. Clinicians' Expectations of the Benefits and Harms of Treatments, Screening, and Tests: A Systematic Review. *JAMA Internal Medicine* 2017;177(3):407-19. doi: 10.1001/jamainternmed.2016.8254
12. Wegwarth O, Gigerenzer G. Overdiagnosis and Overtreatment: Evaluation of What Physicians Tell Their Patients About Screening Harms. *JAMA Internal Medicine* 2013;173(22):2086-88. doi: 10.1001/jamainternmed.2013.10363
13. Seedat F, Cooper J, Cameron L, et al. International comparisons of screening policy-making: A systematic review 2014.

For peer review only

For peer review only - http://bmjopen.bmj.com/site/about/guidelines.xhtml

14. Braithwaite J, Churruca K, Long JC, et al. When complexity science meets implementation science: a theoretical and empirical analysis of systems change. *BMC Medicine* 2018;16(1):63. doi: 10.1186/s12916-018-1057-z

15. Elmore RF. Backward Mapping: Implementation Research and Policy Decisions. *Political Science Quarterly* 1979;94(4):601-16. doi: 10.2307/2149628

16. Noble CH. The Eclectic Roots of Strategy Implementation Research. *Journal of Business Research* 1999;45(2):119-34. doi: https://doi.org/10.1016/S0148-2963(97)00231-2

17. Lanham HJ, Leykum LK, Taylor BS, et al. How complexity science can inform scale-up and spread in health care: understanding the role of self-organization in variation across local contexts. *Social science & medicine (1982)* 2013;93:194-202. doi: 10.1016/j.socscimed.2012.05.040 [published Online First: 2012/07/24]

18. Bauer MS, Damschroder L, Hagedorn H, et al. An introduction to implementation science for the non-specialist. *BMC psychology* 2015;3:32. doi: 10.1186/s40359-015-0089-9 [published Online First: 2015/09/18]

19. Peters DH, Adam T, Alonge O, et al. Implementation research: what it is and how to do it. *BMJ : British Medical Journal* 2013;347:f6753. doi: 10.1136/bmj.f6753

20. Greenhalgh T, Papoutsi C. Studying complexity in health services research: desperately seeking an overdue paradigm shift. *BMC Medicine* 2018;16(1):95. doi: 10.1186/s12916-018-1089-4

21. Long KM, McDermott F, Meadows GN. Being pragmatic about healthcare complexity: our experiences applying complexity theory and pragmatism to health services research. *BMC Medicine* 2018;16(1):94. doi: 10.1186/s12916-018-1087-6

22. Plsek PE, Wilson T. Complexity, leadership, and management in healthcare organisations. *BMJ (Clinical research ed)* 2001;323(7315):746-49. doi: 10.1136/bmj.323.7315.746

23. Cohn S, Clinch M, Bunn C, et al. Entangled complexity: why complex interventions are just not complicated enough. *Journal of health services research & policy* 2013;18(1):40-3. doi: 10.1258/jhsrp.2012.012036 [published Online First: 2013/02/09]

24. Proctor E, Silmere H, Raghavan R, et al. Outcomes for implementation research: conceptual distinctions, measurement challenges, and research agenda. *Administration and policy in mental health* 2011;38(2):65-76. doi: 10.1007/s10488-010-0319-7 [published Online First: 2010/10/20]

25. Davies P, Walker AE, Grimshaw JM. A systematic review of the use of theory in the design of guideline dissemination and implementation strategies and interpretation of the results of rigorous evaluations. *Implementation Science* 2010;5(1):14. doi: 10.1186/1748-5908-5-14

26. Robson J. Screening in general practice and primary care. *British Medical Bulletin* 1998;54(4):961-82. doi: 10.1093/oxfordjournals.bmb.a011741

27. Lai CK, Igarashi A, Lau NM, et al. Cancer screening for older people: to screen or not to screen. *Hong Kong medical journal = Xianggang yi xue za zhi* 2017;23(5):503-16. doi: 10.12809/hkmj166154 [published Online First: 2017/09/02]

28. Shimizu T, Bouchard M, Mavriplis C. Update on age-appropriate preventive measures and screening for Canadian primary care providers. *Can Fam Physician* 2016;62(2):131-38.

29. Emery JD, Shaw K, Williams B, et al. The role of primary care in early detection and follow-up of cancer. *Nature Reviews Clinical Oncology* 2014;11(1):38-48. doi: 10.1038/nrclinonc.2013.212

30. Miser WF. Cancer screening in the primary care setting: the role of the primary care physician in screening for breast, cervical, colorectal, lung, ovarian, and prostate cancers. *Primary care* 2007;34(1):137-67. doi: 10.1016/j.pop.2007.02.002 [published Online First: 2007/05/08]

31. Frame PS, Werth PL. How primary health care providers can integrate cancer prevention into practice. *Cancer* 1993;72(3 Suppl):1132-7. doi: 10.1002/1097-0142(19930801)72:3+<1132::aid-cncr2820721331>3.0.co;2-v [published Online First: 1993/08/01]

32. Kale MS, Korenstein D. Overdiagnosis in primary care: framing the problem and finding solutions. *BMJ (Clinical research ed)* 2018;362:k2820-k20. doi: 10.1136/bmj.k2820

For peer review only

For peer review only - http://bmjopen.bmj.com/site/about/guidelines.xhtml

33. Simonian SJ. Screening and Identification in Pediatric Primary Care. *Behavior Modification* 2006;30(1):114-31. doi: 10.1177/0145445505283311
34. Hamilton W, Walter FM, Rubin G, et al. Improving early diagnosis of symptomatic cancer. *Nature reviews Clinical oncology* 2016;13(12):740-49. doi: 10.1038/nrclinonc.2016.109 [published Online First: 2016/11/03]
35. World Health Organization. Primary Health Care: World Health Organization; 2019 [updated 27/02/2019. Available from: https://www.who.int/news-room/fact-sheets/detail/primary-health-care accessed 27/11/2019.
36. Macinko J, Starfield B, Shi L. The Contribution of Primary Care Systems to Health Outcomes within Organization for Economic Cooperation and Development (OECD) Countries, 1970–1998. *Health Services Research* 2003;38(3):831-65. doi: 10.1111/1475-6773.00149
37. World Health Organization. The World Health Report 2008 - primary Health Care (Now More Than Ever). Geneva, Switzerland: World Health Organization, 2008.
38. Rao M, Pilot E. The missing link--the role of primary care in global health. *Glob Health Action* 2014;7:23693. doi: 10.3402/gha.v7.23693 [published Online First: 2014/02/25]
39. Allen J, Gay B, Crebolder H, et al. The European definition of general practice / family medicine: WONCA Europe, 2005.
40. WHO Regional Office for Europe. Integrated care models: an overview: working document. Copenhagen, Denmark: World Health Organization, 2016.
41. O'Gurek DT, Czapp P, Lampton L, et al. Integration of Primary Care and Public Health (Position Paper): American Academy of Family Physicians, 2015.
42. Baird B. Primary care networks explained: The King's Fund; 2019 [updated 01/07/2019. Available from: https://www.kingsfund.org.uk/publications/primary-care-networks-explained accessed 11/03/2020.
43. Mounier-Jack S, Mayhew SH, Mays N. Integrated care: learning between high-income, and low-and middle-income country health systems. *Health Policy and Planning* 2017;32(suppl_4):iv6-iv12. doi: 10.1093/heapol/czx039
44. Tricco AC, Lillie E, Zarin W, et al. PRISMA Extension for Scoping Reviews (PRISMA-ScR): Checklist and Explanation. *Annals of Internal Medicine* 2018;169(7):467-73. doi: 10.7326/m18-0850
45. Centre for Reviews and Disseminaton. Systematic Reviews: CRD’s guidance for undertaking reviews in health care. York, United Kingdom: Centre for Reviews and Dissemination, University of York, 2009.
46. Noyes J, Booth A, Cargo M, et al. Chapter 21: Qualitative evidence. In: Cochrane Handbook for Systematic Reviews of Interventions version 6.0. In: Higgins J, Thomas J, Chandler J, et al., eds.: Cochrane, 2019.
47. Carroll C, Booth A, Cooper K. A worked example of "best fit" framework synthesis: A systematic review of views concerning the taking of some potential chemopreventive agents. *BMC Medical Research Methodology* 2011;11(1):29. doi: 10.1186/1471-2288-11-29
48. Carroll C, Booth A, Leaviss J, et al. “Best fit” framework synthesis: refining the method. *BMC Medical Research Methodology* 2013;13(1):37. doi: 10.1186/1471-2288-13-37
49. Moher D, Liberati A, Tetzlaff J, et al. Preferred reporting items for systematic reviews and meta-analyses: the PRISMA statement. *BMJ* 2009;339:b2535. doi: 10.1136/bmj.b2535
50. Moher D, Shamseer L, Clarke M, et al. Preferred reporting items for systematic review and meta-analysis protocols (PRISMA-P) 2015 statement. *Systematic reviews* 2015;4(1):1.
51. PROSPERO. PROSPERO: International prospective register of systematic reviews 2020 [Available from: https://www.crd.york.ac.uk/prospero/ accessed 16/03/2020.
52. Scott IA, Attia J, Moynihan R. Promises and perils of using genetic tests to predict risk of disease. *BMJ* 2020;368:m14. doi: 10.1136/bmj.m14
53. WHO Evaluation Office. Review of 40 years of primary health care implementation at country level: World Health Organization, 2019.

For peer review only - http://bmjopen.bmj.com/site/about/guidelines.xhtml

54. The World Bank. World Bank Country and Lending Groups: The World Bank; 2019 [Available from: https://datahelpdesk.worldbank.org/knowledgebase/articles/906519-world-bank-country-and-lending-groups accessed 27/11/2019.
55. Covidence. Covidence: Better systematic review management 2020 [Available from: https://www.covidence.org/home accessed 19/06/2020.
56. Clarivate. Endnote 2020 [Available from: https://endnote.com/14/08/2020.
57. QSR International. NVivo 2020 [Available from: https://www.qsrinternational.com/nvivo-qualitative-data-analysis-software/home accessed 19/06/2020.
58. Hong Q, Pluye P, Fàbregues S, et al. Mixed Methods Appraisal Tool (MMAT), version 2018. Registration of Copyright (#1148552), Canadian Intellectual Property Office, Industry Canada., 2018.
59. Booth A, Carroll C. Systematic searching for theory to inform systematic reviews: is it feasible? Is it desirable? *Health Information & Libraries Journal* 2015;32(3):220-35.
60. Dixon-Woods M, Shaw Rl Fau - Agarwal S, Agarwal S Fau - Smith JA, et al. The problem of appraising qualitative research. (1475-3898 (Print))
61. Petticrew M, Egan M, Thomson H, et al. Publication bias in qualitative research: what becomes of qualitative research presented at conferences? *Journal of Epidemiology and Community Health* 2008;62(6):552-54. doi: 10.1136/jech.2006.059394

FIGURES

Figure 1. Explanation of the role of best fit frameworks in the syntheses for the scoping and systematic reviews on the delivery of screening programmes in primary care.

For peer review only

For peer review only - http://bmjopen.bmj.com/site/about/guidelines.xhtml

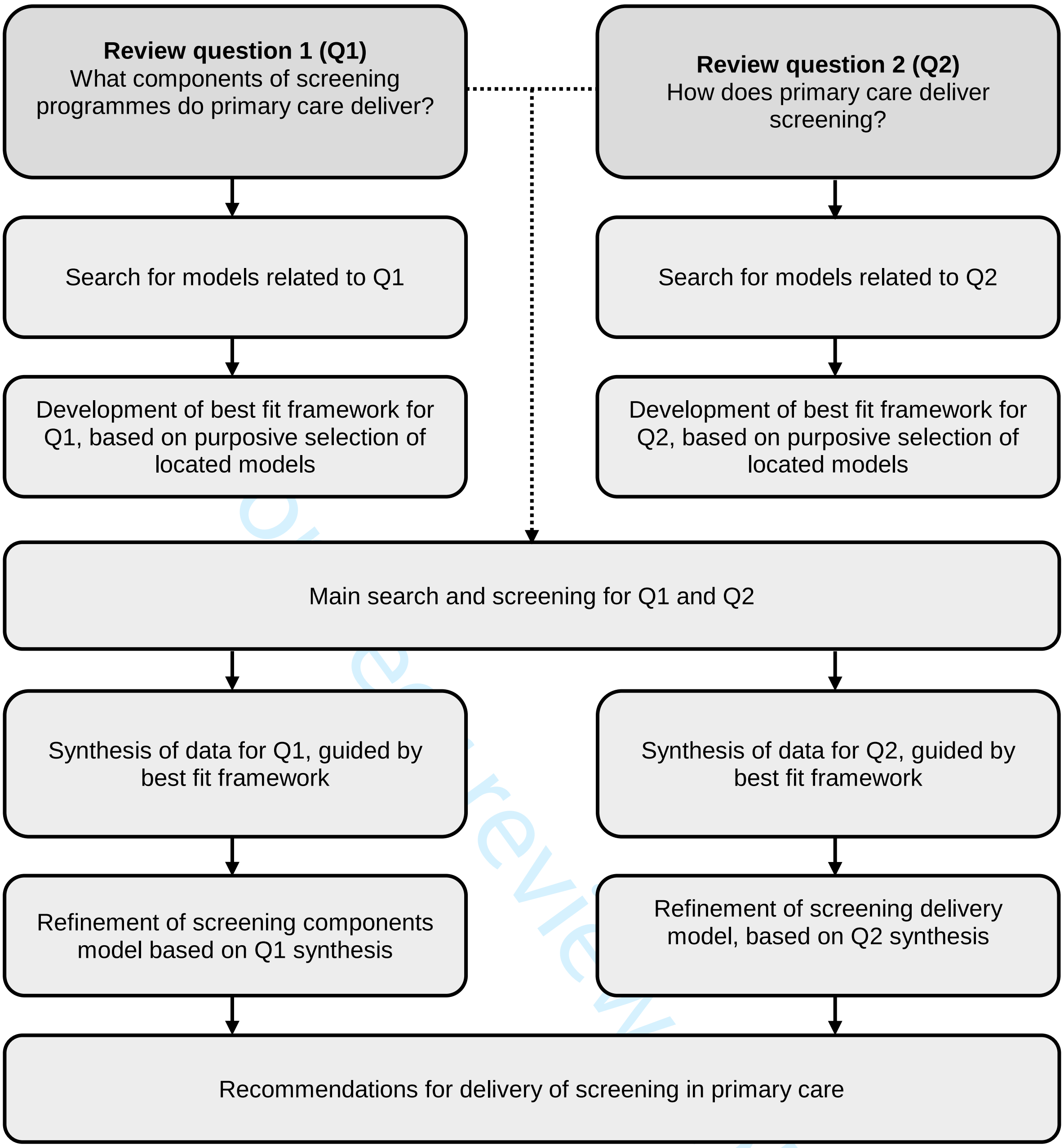
Review question 1 (Q1)
What components of screening programmes do primary care deliver?
Review question 2 (Q2)
How does primary care deliver screening?
Search for models related to Q1
Search for models related to Q2
Development of best fit framework for Q1, based on purposive selection of located models
Development of best fit framework for Q2, based on purposive selection of located models
Main search and screening for Q1 and Q2
Synthesis of data for Q1, guided by best fit framework
Synthesis of data for Q2, guided by best fit framework
Refinement of screening components model based on Q1 synthesis
Refinement of screening delivery model, based on Q2 synthesis
Recommendations for delivery of screening in primary care

Supplementary file 1: Draft search strategy for primary studies, Ovid MEDLINE

Database: Ovid MEDLINE(R) and Epub Ahead of Print, In-Process & Other Non-Indexed Citations, Daily and Versions(R) <1946 to September 14, 2020>

Search Strategy:

----

1 exp Mass Screening/ or exp Early Diagnosis/ or exp "Early Detection of Cancer"/ or exp undiagnosed diseases/

2 (screening or prevention or (undiagnosed or un-diagnosed)).ti. or (early detection or screening test).mp.

3 (piggyback* or opportunis*).ti,ab.

4 1 or 2 or 3

5 Primary Health Care/ or General Practice/ or Family Practice/ or *General Practitioners/ or *Physicians, Family/ or *Community Health Services/ or *Community Pharmacy Services/ or *Pharmacies/ or *Preventive Health Services/

6 ("primary care" or "primary health*" or "General practi*" or "Family practi*" or "family physician*" or "family doctor*").ti,ab.

7 5 or 6

8 (Implement* or behav* or transl* or embed* or Innovat* or Process* or Run* or Manag* or Plan* or improv* or operat* or organis* or organiz* or integrat* or (identif* adj3 eligi*) or pathway* or central* or deliv* or partner* or evaluat* or diffusion* or effect* or effic* or (quality adj3 control*) or enable* or barrier* or facilitat* or strateg* or interven* or (carry adj out)).ti.

9 *Health Plan Implementation/ or *Implementation Science/ or "Outcome and Process Assessment, Health Care"/ or Program Evaluation/ or *Organizational Innovation/ or *"Delivery of Health Care"/ or *Clinical Audit/ or *Management Audit/ or *Quality Control/

10 8 or 9

11 4 and 7 and 10

For peer review only

Supplementary file 2: MMAT checklist from Hong et al. 2018[1]

Part I: Mixed Methods Appraisal Tool (MMAT), version 2018

Category of study designs	Methodological quality criteria	Responses			
		Yes	No	Can't tell	Comments
Screening questions (for all types)	S1. Are there clear research questions?				
	S2. Do the collected data allow to address the research questions?				
	Further appraisal may not be feasible or appropriate when the answer is 'No' or 'Can't tell' to one or both screening questions.				
1. Qualitative	1.1. Is the qualitative approach appropriate to answer the research question?				
	1.2. Are the qualitative data collection methods adequate to address the research question?				
	1.3. Are the findings adequately derived from the data?				
	1.4. Is the interpretation of results sufficiently substantiated by data?				
	1.5. Is there coherence between qualitative data sources, collection, analysis and interpretation?				
2. Quantitative randomized controlled trials	2.1. Is randomization appropriately performed?				
	2.2. Are the groups comparable at baseline?				
	2.3. Are there complete outcome data?				
	2.4. Are outcome assessors blinded to the intervention provided?				
	2.5 Did the participants adhere to the assigned intervention?				
3. Quantitative non-randomized	3.1. Are the participants representative of the target population?				
	3.2. Are measurements appropriate regarding both the outcome and intervention (or exposure)?				
	3.3. Are there complete outcome data?				
	3.4. Are the confounders accounted for in the design and analysis?				
	3.5. During the study period, is the intervention administered (or exposure occurred) as intended?				
4. Quantitative descriptive	4.1. Is the sampling strategy relevant to address the research question?				
	4.2. Is the sample representative of the target population?				
	4.3. Are the measurements appropriate?				
	4.4. Is the risk of nonresponse bias low?				
	4.5. Is the statistical analysis appropriate to answer the research question?				
5. Mixed methods	5.1. Is there an adequate rationale for using a mixed methods design to address the research question?				
	5.2. Are the different components of the study effectively integrated to answer the research question?				
	5.3. Are the outputs of the integration of qualitative and quantitative components adequately interpreted?				
	5.4. Are divergences and inconsistencies between quantitative and qualitative results adequately addressed?				
	5.5. Do the different components of the study adhere to the quality criteria of each tradition of the methods involved?				

1. Hong Q, Pluye P, Fàbregues S, et al. Mixed Methods Appraisal Tool (MMAT), version 2018. Registration of Copyright (#1148552), Canadian Intellectual Property Office, Industry Canada., 2018.

For peer review only

Supplementary file 3: Draft search strategy for best fit framework for research question one, Ovid MEDLINE

Database: Ovid MEDLINE(R) and Epub Ahead of Print, In-Process & Other Non-Indexed Citations, Daily and Versions(R) <1946 to present>

Search Strategy:

----

1 screen*.mp. or exp mass screening/

2 (Pathway* or Process* or Run* or Plan* or Schem* or Blueprint* or Step* or Stage* or Guide*).mp. or exp health plan implementation/ or exp implementation science/ or exp delivery of health care/ [mp=title, abstract, original title, name of substance word, subject heading word, floating sub-heading word, keyword heading word, organism supplementary concept word, protocol supplementary concept word, rare disease supplementary concept word, unique identifier, synonyms]

3 (General practi* or Family practi* or Primary care or Primary health or Family physician* or Family doctor*).mp. or exp General practice/ or exp Primary health care/ or exp Family practice/ or exp General practitioners/ or exp Physicians, family/

4 (pathway* or map or mapping or plan or plans or step or steps or stages or Model* or Theor* or framework* or concept*).mp. or exp Models, theoretical/ or exp Concept formation/

5 1 and 2 and 3 and 4

6 (Regress*model* or integrat* model* or integrat* care model* or economic model* or Markov or Animal or Linear model* or Logistic model* or Likelihood function* or Normogram*).mp. or exp Regression analysis/ or exp Models, statistical/ or exp Markov chains/ or exp Models, chemical/ or exp Models, biological/ or exp Models, molecular/ or exp Fractals/ or exp Metal-organic frameworks/

7 5 not 6

For peer review only

Supplementary file 4: Draft search strategy for best fit framework for research question two, Ovid MEDLINE

Database: Ovid MEDLINE(R) and Epub Ahead of Print, In-Process & Other Non-Indexed Citations, Daily and Versions(R) <1946 to present>

Search Strategy:

----

1 Screen*.mp. or exp mass screening/

2 (Implement* or deliver* or translat* or integrat* or Innovat* or Manag*).mp. or exp Health plan implementation/ or exp Implementation science/ or exp Organizational innovation/ or exp Diffusion of innovation/ or exp Delivery of health care/

3 (General practi* or Family practi* or Primary care or Primary health or Family physician* or Family doctor*).mp. or exp General practice/ or exp Primary health care/ or exp Family practice/ or exp General practitioners/ or exp Physicians, family/

4 (Model* or Theor* or framework* or concept*).mp. or exp Models, theoretical/ or exp Concept formation/

5 1 and 2 and 3 and 4

6 (Regress*model* or integrat* model* or integrat* care model* or economic model* or Markov or Animal or Linear model* or Logistic model* or Likelihood function* or Normogram*).mp. or exp Regression analysis/ or exp Models, statistical/ or exp Markov chains/ or exp Models, chemical/ or exp Models, biological/ or exp Models, molecular/ or exp Fractals/ or exp Metal-organic frameworks/

7 5 not 6

For peer review only

PRISMA-P (Preferred Reporting Items for Systematic review and Meta-Analysis Protocols) 2015 checklist: recommended items to address in a systematic review protocol*

Section and topic	Item No	Checklist item	
**ADMINISTRATIVE INFORMATION**			
Title:			
Identification	1a	Identify the report as a protocol of a systematic review	title page
Update	1b	If the protocol is for an update of a previous systematic review, identify as such	N/A
Registration	2	If registered, provide the name of the registry (such as PROSPERO) and registration number	Abstract
Authors:			
Contact	3a	Provide name, institutional affiliation, e-mail address of all protocol authors; provide physical mailing address of corresponding author	Title page
Contributions	3b	Describe contributions of protocol authors and identify the guarantor of the review	p12
Amendments	4	If the protocol represents an amendment of a previously completed or published protocol, identify as such and list changes; otherwise, state plan for documenting important protocol amendments	p11
Support:			
Sources	5a	Indicate sources of financial or other support for the review	p12
Sponsor	5b	Provide name for the review funder and/or sponsor	p12
Role of sponsor or funder	5c	Describe roles of funder(s), sponsor(s), and/or institution(s), if any, in developing the protocol	p12
**INTRODUCTION**			
Rationale	6	Describe the rationale for the review in the context of what is already known	p1-2
Objectives	7	Provide an explicit statement of the question(s) the review will address with reference to participants, interventions, comparators, and outcomes (PICO)	p2 (PICO format not appropriate)
**METHODS**			
Eligibility criteria	8	Specify the study characteristics (such as PICO, study design, setting, time frame) and report characteristics (such as years considered, language, publication status) to be used as criteria for eligibility for the review	p3-6
Information sources	9	Describe all intended information sources (such as electronic databases, contact with study authors, trial registers or other grey literature sources) with planned dates of coverage	

For peer review only

			p6-7
Search strategy	10	Present draft of search strategy to be used for at least one electronic database, including planned limits, such that it could be repeated	p7-8 and supplementary file 1
Study records:			
Data management	11a	Describe the mechanism(s) that will be used to manage records and data throughout the review	p8
Selection process	11b	State the process that will be used for selecting studies (such as two independent reviewers) through each phase of the review (that is, screening, eligibility and inclusion in meta-analysis)	p8
Data collection process	11c	Describe planned method of extracting data from reports (such as piloting forms, done independently, in duplicate), any processes for obtaining and confirming data from investigators	p8-9
Data items	12	List and define all variables for which data will be sought (such as PICO items, funding sources), any pre-planned data assumptions and simplifications	p8-9
Outcomes and prioritization	13	List and define all outcomes for which data will be sought, including prioritization of main and additional outcomes, with rationale	p9
Risk of bias in individual studies	14	Describe anticipated methods for assessing risk of bias of individual studies, including whether this will be done at the outcome or study level, or both; state how this information will be used in data synthesis	p9 and supplementary file 2
Data synthesis	15a	Describe criteria under which study data will be quantitatively synthesised	p9-11
	15b	If data are appropriate for quantitative synthesis, describe planned summary measures, methods of handling data and methods of combining data from studies, including any planned exploration of consistency (such as I^2, Kendall's τ)	N/A
	15c	Describe any proposed additional analyses (such as sensitivity or subgroup analyses, meta-regression)	p11
	15d	If quantitative synthesis is not appropriate, describe the type of summary planned	p9-11
Meta-bias(es)	16	Specify any planned assessment of meta-bias(es) (such as publication bias across studies, selective reporting within studies)	p11
Confidence in cumulative evidence	17	Describe how the strength of the body of evidence will be assessed (such as GRADE)	p11

*** It is strongly recommended that this checklist be read in conjunction with the PRISMA-P Explanation and Elaboration (cite when available) for important clarification on the items. Amendments to a review protocol should be tracked and dated. The copyright for PRISMA-P (including checklist) is held by the PRISMA-P Group and is distributed under a Creative Commons Attribution Licence 4.0.**

For peer review only

From: Shamseer L, Moher D, Clarke M, Ghersi D, Liberati A, Petticrew M, Shekelle P, Stewart L, PRISMA-P Group. Preferred reporting items for systematic review and meta-analysis protocols (PRISMA-P) 2015: elaboration and explanation. BMJ. 2015 Jan 2;349(jan02 1):g7647.

For peer review only
